# Enhancing Photostability of Complex Lead Halides through Modification with Antibacterial Drug Octenidine

**DOI:** 10.3390/ma17010129

**Published:** 2023-12-26

**Authors:** Victoria V. Ozerova, Ivan S. Zhidkov, Nikita A. Emelianov, Denis V. Korchagin, Gennady V. Shilov, Fedor A. Prudnov, Igor V. Sedov, Ernst Z. Kurmaev, Lyubov A. Frolova, Pavel A. Troshin

**Affiliations:** 1 Federal Research Center for Problems of Chemical Physics and Medicinal Chemistry of the Russian Academy of Sciences, 1 prosp. Semenova, 142432 Chernogolovka, Russia; podsolnuz@gmail.com (V.V.O.); nikita_emelyanov@bk.ru (N.A.E.); korden@icp.ac.ru (D.V.K.); genshil@icp.ac.ru (G.V.S.); prudnov2014@gmail.com (F.A.P.); isedov@icp.ac.ru (I.V.S.); 2 Institute of Physics and Technology, Ural Federal University, 19 ul. Mira, 620002 Yekaterinburg, Russiaernst.kurmaev@gmail.com (E.Z.K.); 3 M. N. Mikheev Institute of Metal Physics of Ural Branch of Russian Academy of Sciences, 18 ul. S. Kovalevskoi, 620108 Yekaterinburg, Russia; 4 Zhengzhou Research Institute, Harbin Institute of Technology, Longyuan East 7th 26, Jinshui District, Zhengzhou 450003, China

**Keywords:** perovskite solar cells, complex lead halides, photostability, molecular additives, molecular modifiers, 2D/3D hybrid perovskites

## Abstract

The high power-conversion efficiencies of hybrid perovskite solar cells encourage many researchers. However, their limited photostability represents a serious obstacle to the commercialization of this promising technology. Herein, we present an efficient method for improving the intrinsic photostability of a series of commonly used perovskite material formulations such as MAPbI_3_, FAPbI_3_, Cs_0.12_FA_0.88_PbI_3_, and Cs_0.10_MA_0.15_FA_0.75_PbI_3_ through modification with octenidine dihydroiodide (***OctI_2_***), which is a widely used antibacterial drug with two substituted pyridyl groups and two cationic centers in its molecular framework. The most impressive stabilizing effects were observed in the case of FAPbI_3_ and Cs_0.12_FA_0.88_PbI_3_ absorbers that were manifested in significant suppression or even blocking of the undesirable perovskite films’ recrystallization and other decomposition pathways upon continuous 110 mW/cm^2^ light exposure. The achieved material photostability—within 9000 h for the Oct(FA)_n−1_Pb_n_I_3n+1_ (n = 40–400) and 20,000 h for Oct(Cs_0.12_FA_0.88_)_n−1_Pb_n_I_3n+1_ (where n = 40–400) formulations—matches the highest values ever reported for complex lead halides. It is important to note that the stabilizing effect is maintained when ***OctI_2_*** is used only as a perovskite surface-modifying agent. Using a two-cation perovskite composition as an example, we showed that the performances of the solar cells based on the developed Oct(Cs_0.12_FA_0.88_)_399_Pb_400_I_1201_ absorber material are comparable to that of the reference devices based on the unmodified perovskite composition. These findings indicate a great potential of the proposed approach in the design of new highly photostable and efficient light absorbers. We believe that the results of this study will also help to establish important guidelines for the rational material design to improve the operational stability of perovskite solar cells.

## 1. Introduction

Organic–inorganic hybrid lead halide perovskites are promising photoactive materials for perovskite solar cells (PSCs), which deliver light power conversion efficiencies over 26% that make this technology very attractive for commercialization [1,2,3]. Unfortunately, these light-absorbing materials exhibit insufficient stability toward heating, moisture, and solar light [4,5,6,7,8,9,10]. The latter is the most critical challenge, as it is the factor that we cannot avoid under the solar cell operational conditions.

One approach to improve PCS durability is the introduction of bulk cations into the three-dimensional structure of APbI_3_ perovskite with a small univalent cation, A, such as Cs^+^, MA^+^ (CH_3_NH_3_^+^), and FA^+^ (CH(NH_2_)^2+^) thus resulting in layered 2D/3D materials, which can be represented by the general formula (A′)_m_(A)_n−1_Pb_n_I_3n+1_, where A′ is a divalent (m = 1) or monovalent (m = 2) cation. These two types of materials have different crystal structures, where monovalent and divalent organic cations form, respectively, double and single interlayers between the 3D perovskite layers and are classified as Ruddlesden–Popper and Dion–Jacobson perovskite-like phases. The 3D perovskite layer size is defined by the n value and can be controlled by tuning the precursor composition, whereas the interlayer distance is depending on the molecular structure of the spacer cations [11,12].

To date, the most intensive studies have focused on Ruddlesden–Popper phases (A′_2_A_n−1_Pb_n_I_3n+1_) based on bulk monovalent cations such as a phenylethylamine (PEA) derivatives [13,14,15], n-butylamine (BA) [16], and others [17] which are able to passivate defects and improve the ambient stability of the materials. For example, the modified Ruddlesden–Popper-type films could tolerate high air humidity conditions due to the hydrophobic nature of the bulk organic cation A’ and dense crystal packing [18,19]. In addition, ion migration is suppressed in quasi-2D perovskite materials due to higher activation energies for the formation of point defects [20].

The introduction of divalent organic cations instead of monovalent (such as ethylene diammonium [21], N,N′-dimethylethylene diammonium [22], 1,8-octamethylenediammonium [23], 1,4-butanediammonium [24], 1,4-phenylenedimethanammonium [25] etc. [26]) seems to be more promising than designing similar Ruddlesden–Popper structures in terms of achieving higher structural stability, since divalent bulk cations directly connect the neighboring 3D perovskite layers by electrostatic coulombic interactions and also hydrogen bonds. On the contrary, the alternating 3D layers in the Ruddlesden–Popper phases are held together only via the weak van der Waals forces [27].

In addition, Dion–Jacobson perovskite-like phases demonstrate shorter interlayer distances with stronger interactions between PbI_6_ octahedrons layers, which might enable enhanced charge transport, and accelerate charge separation and extraction [28,29,30]. Although the Dion–Jacobson phases represent very promising light-absorbing materials owing to their higher structural stability as compared to the Ruddlesden–Popper counterparts, they are currently much less studied [31,32,33].

When developing Dion–Jacobson phases, researchers face a common problem of low-dimensional perovskites such as wide bandgaps of 2D materials, large exciton binding energies and low charge mobility, which seriously limits their applicability in photovoltaics. The optical bandgap and electronic properties of low dimensional A′(A)_n−1_Pb_n_I_3n+1_ perovskites are significantly influenced by the size of the perovskite 3D layers (n-value). With an increase in the *n* value, the electronic bands broaden, which weakens quantum confinement and promotes a narrower bandgap [34]. Some reports have shown a direct connection between the value of n and the short-circuit current density (*J*_SC_) in solar cells based on 2D perovskites [31,35,36,37]. For instance, light absorbers based on Dion–Jacobson phases comprising 1,4-phenylenedimethylammonium cations provide an increase in the solar cells’ current density from 11.5 to 21.5 mA/cm^2^ as the value of n evolves from 3 to 10 [38,39]. Furthermore, the increase in n value can result in better alignment of the semiconductor valence and conduction band positions with the frontier energy levels of, e.g., organic charge transport layer materials as revealed for Dion–Jacobson systems comprising cations derived from 1,4-butanediamine (BDA) 1,3-propanediamine (PDA), and 1,5-pentamethylenediamine (PeDA) [29,37,40].

Conversely, in the case of all-inorganic systems, the introduction of bulk organic cations can be used to achieve a narrower band gap. The incorporation of ethylenediammonium cations (EDA^2+^) in 3D α-CsPbI_3_ produces (EDA)Cs_n−1_Pb_n_I_3n+1_ (1 < n < 8) compounds, which show decrease in the bandgap upon increase in the n value [41]. Furthermore, the optimal (EDA)Cs_n−1_Pb_n_I_3n+1_ absorbers demonstrated improved charge carrier generation and transport as compared to 3D α-CsPbI_3_.

The chemical structure of organic cations has a strong influence on the optoelectronic properties and structure (e.g., interlayer distance) of the 2D perovskites, their charge dynamics and photovoltaic performance [42]. Until now, various aliphatic and aromatic alkylammonium cations have been investigated to improve the characteristics of the Dion–Jacobson perovskites (overview of the literature data is given in the Table S1, Supplementary Materials) [21,22,24,25,29,30,31,32,36,38,39,40,42,43,44,45,46,47,48,49,50,51,52,53,54,55,56,57,58,59,60,61,62,63,64,65,66,67,68,69].

The 2D/3D perovskites incorporating bulky organic (di)cations with conjugated structures emerge as highly promising absorber materials [70,71]. The conjugated aromatic cations have higher dielectric constants as compared to aliphatic ones, which helps to reduce the binding energy of excitons and thus enhance the photogeneration of charge carriers [29,30,39]. For instance, the use of ampicillin as the spacer cation has been reported to improve the efficiency of light emitting diodes due to the better Fermi level alignment, improved charge balance and reduced leakage losses [72,73].

Notably, the vast majority of research is aimed at identifying suitable organic cations to form 2D/3D perovskites with desired properties, in particular the enhanced hydrophobicity resulting in their superior resistance to hydrolysis. However, the actual practical use of PSCs will require encapsulation that provides an efficient barrier to oxygen and moisture. Therefore, the improvement of the ambient stability of light-absorbing materials is not as important as enhancing their intrinsic stability with respect to light, since this is the stress factor that we cannot avoid under real solar cell operational conditions [74]. However, the highest lifetimes reported so far for PSCs based on the Dion–Jacobson materials is much lower than required for their practical application (Table S1, Supplementary Materials). Consequently, it is an urgent task to further increase the photostability of the Dion–Jacobson perovskites and the corresponding solar cells.

Herein, we present an efficient method to improve the photostability of a series of the most commonly used perovskite compositions such as MAPbI_3_, FAPbI_3_, Cs_0.12_FA_0.88_PbI_3_, and Cs_0.10_MA_0.15_FA_0.75_PbI_3_ by the incorporation of a divalent octenidine cation with a bulky conjugated molecular structure into the 3D perovskite crystal lattice to form 2D/3D material formulations. The modifier used, octenidine dihydroiodide (***OctI_2_***), is related to the widely used antibacterial drug octenidine dihydrochloride [75,76,77,78]. We have shown that the proposed optimally modified light absorbers exhibit record photostability and competitive photovoltaic performances in perovskite solar cells. This work may provide important insights into the rational design of perovskite light absorbers with largely enhanced stability through compositional and structural engineering, thus opening broad new opportunities for the fabrication of efficient and durable PSCs.

## 2. Materials and Methods

### 2.1. Materials

Formamidinium iodide (FAI, purity 99.9%), methylammonium iodide (MAI, purity 99.9%), phenyl-C_61_-butyric acid methyl ester (PCBM purity 99.99%), and poly[bis(4-phenyl)(4-methylphenyl)amine] (PTA, 99.9%) were purchased from FOMaterials Ltd., (Moscow, Russia). Cesium iodide (CsI, purity 99.999%) and lead iodide (PbI_2_, purity 99.99%) were acquired from Aldrich (Saint Louis, MO, USA) and Lanhit (Moscow, Russia), respectively. Sodium iodide (NaI, purity 99%) was purchased from Acros (Geel, Belgium).

Octenidine dihydroiodide (N,N′-(1,10-decanediyldi-1(4H)-pyridinyl-4-ylidene)bis-1-octanamine dihydroiodide) (***OctI_2_***) was synthesized using the reaction between octenidine dihydrochloride and NaI in methanol as described previously [31]. Anhydrous dimethylformamide (DMF), dimethylacetamide (DMAc), dimethyl sulfoxide (DMSO), N-methyl-2-pyrrolidone (NMP), toluene, chlorobenzene and dichloromethane were purchased from Sigma-Aldrich and used as received inside nitrogen glove boxes. Methanol (99.9%, for analysis) was acquired from Acros Organics (Belgium). Glass slides (25 × 25 mm) were cut from standard objective borosilicate glass purchased from Isolab GmbH. Indium–tin-oxide (ITO)-coated glass substrates (15 Ω sq^−1^) were acquired from the Kintec Company (Changan Town, China).

### 2.2. Preparation of the Perovskite Films

#### 2.2.1. Cleaning of Substrates

The glass or ITO substrates were cleaned sequentially with deionized water, acetone, and isopropyl alcohol by sonication for 15 min followed by the RF-air plasma treatment for 5 min. All further manipulations were performed under a well-controlled atmosphere inside nitrogen glove boxes.

#### 2.2.2. Preparation of Perovskite Precursor Solutions

A 1.35 M MAPbI_3_ precursor solution was prepared by stoichiometrically mixing MAI and PbI_2_ in an anhydrous DMF. The Oct(MA)_39_Pb_40_I_121_ (or Oct(MA)_399_Pb_400_I_1201_) precursor solutions were obtained by dissolving 0.0338 (or 0.0034) mmol of ***OctI_2_***, 1.3163 (or 1.3466) mmol of MAI, and 1.35 mmol of PbI_2_ in 1 mL of anhydrous DMF under stirring at 70 °C.

A FAPbI_3_ precursor solution with a concentration of 1.3 M was obtained by stoichiometrically mixing FAI and PbI_2_ in anhydrous DMAc:NMP (1:1 *v*/*v*) mixture. The Oct(FA)_39_Pb_40_I_121_ (and Oct(FA)_399_Pb_400_I_1201_) precursor solutions were obtained by dissolving 0.0325 (and 0.0033) mmol of ***OctI_2_***, 1.2675 (and 1.2968) mmol of FAI, and 1.3 mmol of PbI_2_ in 1 mL of anhydrous DMAc:NMP (1:1 *v*/*v*) mixture under stirring at room temperature.

A Cs_0.12_FA_0.88_PbI_3_ precursor solution with a concentration of 1.3 M was obtained by dissolving together 0.156 mmol of CsI, 1.144 mmol of FAI, and 1.3 mmol of PbI_2_ in 1.0 mL of anhydrous DMAc:DMSO (9:1 *v*/*v*) mixture under continuous stirring at room temperature. The Oct(Cs_0.12_FA_0.88_)_39_Pb_40_I_121_ (and Oct(Cs_0.12_FA_0.88_)_399_Pb_400_I_1201_) precursor solutions were obtained by dissolving 0.0325 (0.0033) mmol of ***OctI_2_***, 0.1521 (0.1556) mmol of CsI, 1.1154 (1.1411) mmol of FAI, and 1.300 mmol of PbI_2_ in 1.0 mL of an anhydrous DMAc:DMSO (9:1 *v*/*v*) mixture under continuous stirring at room temperature.

A Cs_0.1_MA_0.15_FA_0.75_PbI_3_ precursor solution with a concentration of 1.4 M was obtained by dissolving together 0.14 mmol of CsI, 0.21 mmol of MAI, 1.05 mmol of FAI, and 1.4 mmol of PbI_2_ in 1 mL of anhydrous DMF:DMSO (4:1 *v*/*v*) mixture under continuous stirring at room temperature. The Oct(Cs_0.1_MA_0.15_FA_0.75_)_39_Pb_40_I_121_ (and Oct(Cs_0.1_MA_0.15_FA_0.75_)_399_Pb_400_I_1201_) precursor solutions were obtained by dissolving 0.035 (0.0035) mmol of ***OctI_2_***, 0.1365 (0.1397) mmol of CsI, 0.2048 (0.2095) mmol of MAI, 1.0238 (1.0474) mmol of FAI, and 1.4 mmol of PbI_2_ in 1 mL of anhydrous DMF:DMSO (4:1 *v*/*v*) mixture under stirring at room temperature.

After complete dissolution, all prepared solutions were filtered through 0.45 µm PTFE membrane syringe filters.

#### 2.2.3. Perovskite Films Deposition

The 45 µL aliquots of the corresponding precursor solutions were dropped on glass substrates rotating at 3000 rpm and then quenched after 8–9 s by dripping 120 µL of anhydrous chlorobenzene in the case of MAPbI_3_, Oct(MA)_39_Pb_40_I_121_ and Oct(MA)_399_Pb_400_I_1201_ film deposition. The samples were kept at 3000 rpm for an additional 40 s and then annealed at 100 °C on a hot plate for 5 min.

To deposit FAPbI_3_, Oct(FA)_39_Pb_40_I_121_ and Oct(FA)_399_Pb_400_I_1201_ films, the 55 µL aliquots of the corresponding precursor solutions were dropped on glass substrates rotating at 3600 rpm and then quenched after 30 s by dripping 120 µL of anhydrous toluene. The samples were kept at 3600 rpm for additional 10 s and then annealed on a hot plate at 90 °C for 2 min and 135–145 °C for 30 min.

In the case of Cs_0.12_FA_0.88_PbI_3_, Oct(Cs_0.12_FA_0.88_)_39_Pb_40_I_121_ and Oct(Cs_0.12_FA_0.88_)_399_Pb_400_I_1201_ film deposition, the 55 µL aliquots of the corresponding precursor solution were dropped on glass substrates rotating at 3600 rpm and then quenched after 20 s by dripping 120 µL of anhydrous toluene. The samples were kept at 3600 rpm for additional 15 s and then annealed on a hot plate at 100 °C for 5 min.

To prepare the Cs_0.1_MA_0.15_FA_0.75_PbI_3_, Oct(Cs_0.1_MA_0.15_FA_0.75_)_39_Pb_40_I_121_ and Oct(Cs_0.1_MA_0.15_FA_0.75_)_399_Pb_400_I_1201_ films, 55 µL aliquots of the corresponding precursor solutions were dropped on the glass substrates rotating at 4000 rpm and then quenched after 29 s by dripping 90 µL of anhydrous chlorobenzene; the samples were kept at 4000 rpm for an additional 21 s and then annealed at 100 °C for 5 min on a hot plate.

All aforementioned manipulations were performed in a well-controlled inert atmosphere inside nitrogen glove boxes.

#### 2.2.4. Post-Treatment of Perovskite Films with ***OctI_2_***

***OctI_2_*** solution with a concentration of 5 mM was obtained by dissolving 4.035 mg of ***OctI_2_*** in anhydrous dichloromethane:chlorobenzene (9:1 *v*/*v*) mixture. For the surface modification of the perovskite films by the post-treatment method, an aliquot of 20 µL of ***OctI_2_*** solution was deposited on the preformed perovskite films by a dynamic spin-coating method at 3000 rpm for 30 s. The resulting samples were then annealed on a hot plate at 100 °C for 5 min.

### 2.3. Photostability Testing

The aging experiments were performed using special home-made setups installed inside MBraun glove boxes under an inert atmosphere of pure nitrogen (concentration of O_2_ and H_2_O < 1 ppm) under continuous light soaking. The white light with the incident power of 70 ± 10 mW/cm^2^ was provided by LED arrays. An assessment of the emission spectrum of LEDs and the absorption profiles of the perovskite films revealed that the absorbed dose of photons by the samples under these conditions was equivalent to the dose provided by ~110 mW/cm^2^ standard AM1.5G solar light irradiation. The temperature of the samples was within 38 ± 3 °C. More details on the aging setup design can be found in our previous publication [79].

### 2.4. Perovskite Film Characterization

The UV–Vis absorption spectra were obtained using an AvaSpec-2048-2 UV–VIS fiber spectrometer integrated inside a glove box. The X-ray diffraction (XRD) patterns were collected using an Aeris instrument (Malvern PANalytical B.V., Malvern, UK) with a CuKα source. The samples for XRD measurements were protected by spin-coating polystyrene films inside the glove box to avoid moisture-induced aging during the measurement in air (~5 min). The photoluminescence (PL) spectra were measured in an inert atmosphere using an Ocean Insight QE Pro spectrometer with a 450 nm laser as an excitation source. The atomic force microscopy (AFM) and infrared scattering-type near-field optical microscopy (IR s-SNOM) measurements were performed using a Neaspec instrument (Munich, Germany, 2020) integrated inside the glove box. The VIT_P/Pt cantilevers (NT-MDT) with Pt tip coating, a probe radius of 25–35 nm, a typical resonance frequency of around 300 kHz and a force constant of 50 N/m were used for the measurements.

Survey and core-level X-ray photoelectron spectra (XPS) were measured using a PHI XPS Versaprobe 500 spectrometer (ULVAC-Physical Electronics, Chanhassen, MN, USA) with a spherical quartz monochromator and an energy analyzer working in the range of binding energies from 0 to 1500 eV. The samples were transferred to the instrument under ambient conditions within 1 min and then kept in a vacuum chamber for 24 h prior to the experiments. The measurements were performed at a pressure of 10^−7^ Pa.

### 2.5. Device Fabrication and Characterization

The ITO glass substrates were cleaned as described above. A solution of PTA in chlorobenzene (2.5 mg mL^−1^) was spin-coated on the ITO substrates at 3500 rpm for 30 s. The samples were then annealed at 120 °C for 15 min inside the glove box. Modified Oct(Cs_0.12_FA_0.88_)_n−1_Pb_n_I_3n+1_ (where n = 400, 200, and 40) formulations were used as light-absorbing layer materials and compared to the Cs_0.12_FA_0.88_PbI_3_ reference. The perovskite films were deposited by spin-coating precursor solutions at 4000 rpm for 80 s. The crystallization was induced by dripping 130 µL of chlorobenzene onto the film at the 36th second after starting the spin-coating process. The obtained perovskite films were annealed at 110 °C for 3 min. A PC_61_BM precursor solution (30 mg mL^−1^ in chlorobenzene) was spin-coated on the top of the perovskite films at 1500 rpm for 30 s. Finally, the Ag or Al electrodes (120 nm) were evaporated through a shadow mask in high vacuum (6 × 10^−6^ Torr) to produce devices with an active area of 0.3 cm^2^. The solar cells were characterized by current–voltage (I–V) and external quantum efficiency (EQE) measurements as reported previously [80].

## 3. Results and Discussion

**Figure 1:**
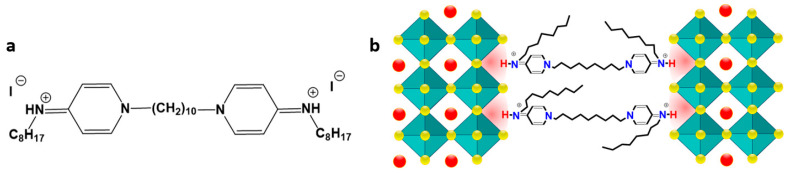
Molecular structure of octenidine dihydroiodide (**a**) and a schematic illustration of the Dion–Jacobson phase that could be formed as a result of ***OctI_2_*** integration into crystal lattice (**b**).

To obtain the 2D/3D perovskites, ***OctI_2_*** was incorporated into precursor solutions of the most commonly used single cation and mixed cation perovskites APbI_3_ (where A = MA, FA, Cs_0.12_FA_0.88_, Cs_0.1_MA_0.15_FA_0.75_) to form the Oct(A)_n−1_Pb_n_I_3n+1_ (where n = 40 and 400) formulations. All perovskite films were fabricated via the single-step spin coating process followed by annealing in a glove box filled with N_2_. Details can be found in the Section 2. The molecular structure of octenidine dihydroiodide and a schematic representation of the Dion–Jacobson phase formed using the ***OctI_2_*** additive are presented in Figure 1.

In order to study the impact of octenidine dihydroiodide on the phase composition and optoelectronic properties of the Oct(A)_n−1_Pb_n_I_3n+1_ (where n = 40 and 400) compositions and monitor their aging behavior, the obtained perovskite films were characterized by UV–Vis absorption spectroscopy and x-ray diffraction before and after exposure to white light (for details, see the Section 2) in a pure nitrogen atmosphere (O_2_ and H_2_O < 1 ppm). Additional insights were provided by using other complementary methods such as X-ray photoelectron spectroscopy, atomic force microscopy, and infrared scattering-type near-field optical microscopy.

**Figure 2:**
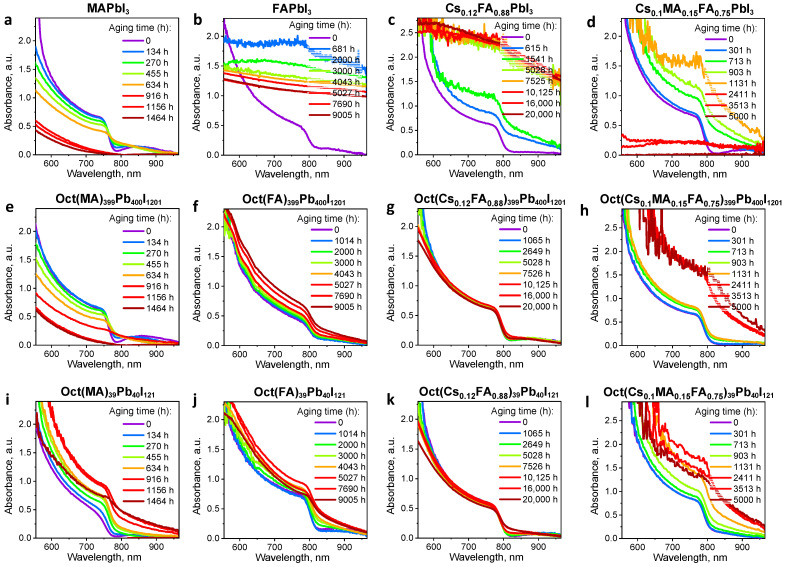
Evolution of the UV–Vis spectra of the Oct(A)_n−1_Pb_n_I_3n+1_ films depending on the ***OctI_2_*** loading as compared to the reference APbI_3_ samples, where A = MA (**a**,**e**,**i**), FA (**b**,**f**,**j**), Cs_0.12_FA_0.88_ (**c**,**g**,**k**), and Cs_0.1_MA_0.15_FA_0.75_ (**d**,**h**,**l**) as a function of the aging time.

Figure 2 shows the UV−Vis absorption spectra of the as-prepared Oct(A)_n−1_Pb_n_I_3n+1_ films and the reference APbI_3_ samples and their evolution depending on the light exposure duration. Absorption coefficients (k) of the pristine and modified absorber films as a function of the wavelength are given in Figure S1 (Supplementary Materials). The first thing to note is that the introduction of octenidine into all studied perovskite systems leads to a change in the optical properties of the Oct(A)_n−1_Pb_n_I_3n+1_ films. All obtained Oct(A)_399_Pb_400_I_1201_ films demonstrate higher absorption coefficients than the corresponding non-modified perovskites. On the contrary, the studied Oct(A)_39_Pb_40_I_121_ formulations with higher octenidine loading demonstrate reduced k values except for Oct(FA)_39_Pb_40_I_121_ films, which showed a noticeable increase in light absorption in the 700–800 nm region.

Figure S2 and Table S2 (Supplementary Materials) show the values of optical absorption edge for as-prepared samples depending on the ***OctI_2_*** loading estimated from the Tauc plots. With increase in the ***Oct*** content in the Oct(A)_n−1_Pb_n_I_3n+1_ films, the optical absorption bands showed noticeable blue shifts in the case of A = MA, Cs_0.12_FA_0.88_ and the red shifts in the case of A = FA, Cs_0.1_MA_0.15_FA_0.75_. The observed different effects of octenidine loading on the optical properties of the resulting perovskite films can result from a combination of several factors. On the one hand, the introduction of bulky organic dication salt ***OctI_2_*** might led to the formation of the Dion–Jacobson (2D/3D) phases, which are characterized by increased band gaps in comparison with the original 3D systems [10,11]. On the other hand, the accumulation of bulky cations mostly on the surface of the perovskite grains and at the grain boundaries was frequently reported [81]. Therefore, the surface and interface effects of ***OctI_2_*** can lead to the red shift of the absorption band edge [82]. In addition, the effect of ***OctI_2_*** loading on the optical properties of the perovskite films could be connected to the change in their nanoscale morphology, e.g., the formation of much bigger or smaller grains than in the reference non-modified systems [83].

Atomic force microscopy showed that the introduction of ***OctI_2_*** markedly changes the absorber layer morphology (Figure S3, Supplementary Materials). Figure S4 (Supplementary Materials) shows the grain size distribution of Oct(A)_39_Pb_40_I_121_ (where A = MA and Cs_0.1_MA_0.15_FA_0.85_) perovskite films extracted from the topography data. For example, the MAPbI_3_ films are characterized by an average crystallite size of 110 nm, whereas Oct(MA)_39_Pb_40_I_121_ exhibited smaller grains (~50 nm) (Figures S3a,b and S4a,b, Supplementary Materials). The grains with a loose structure (~250 nm) are observed in the case of the reference mixed-cation Cs_0.1_MA_0.15_FA_0.85_PbI_3_ perovskite. In comparison, the Oct(Cs_0.1_MA_0.15_FA_0.85_)_39_Pb_40_I_121_ films demonstrate distinct grains with well-defined boundaries and a smaller average size of ~180 nm (Figures S3c,d and S4c,d, Supplementary Materials). We attribute the decrease in the average grain size in the modified films to the possible formation of some intermediate complexes between octenidine iodide and the components of the perovskite precursor solution owing to the strong co-ordinating bonding and hydrogen bonding [84,85]. The modifier accelerates the crystallization process, thus increasing the number of nucleation centers and producing uniform continuous films with the smaller size of the grains.

**Figure 3:**
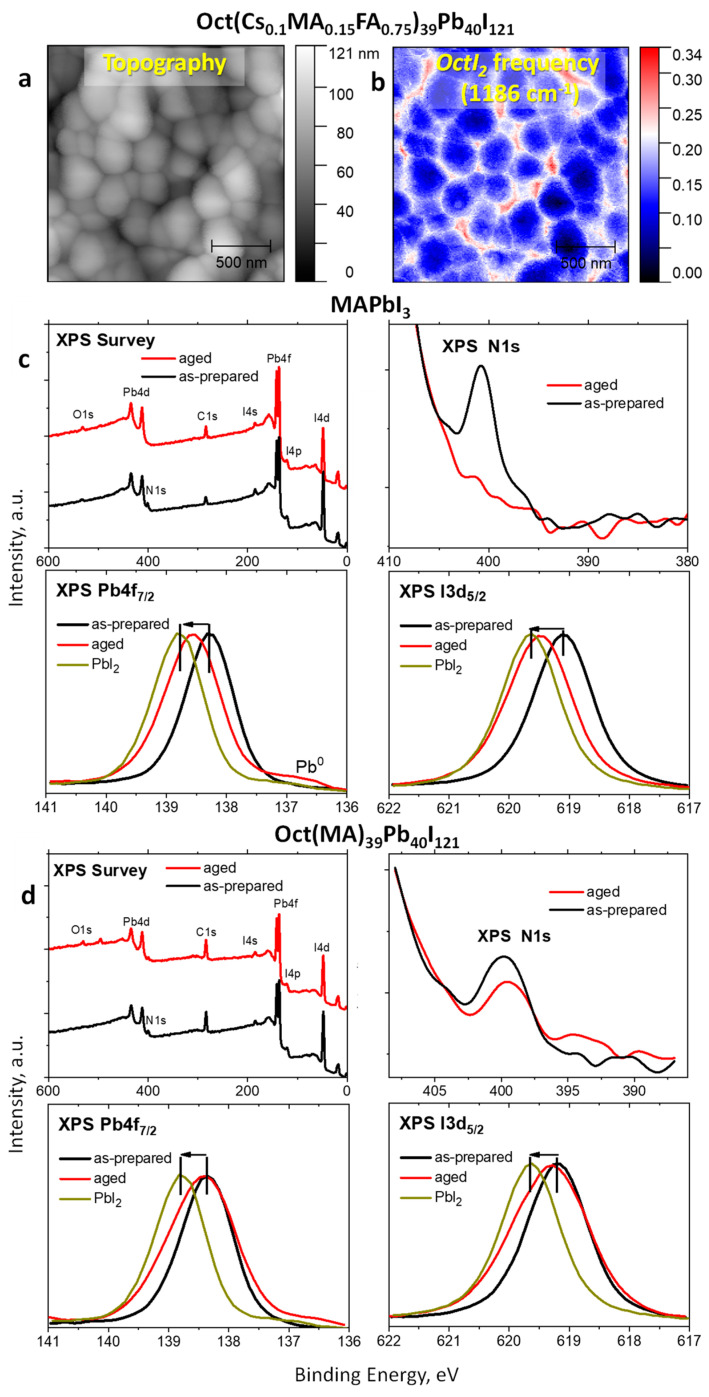
AFM topography (**a**) and s-SNOM amplitude images for the Oct(Cs_0.1_MA_0.15_FA_0.75_)_39_Pb_40_I_121_ film recorded at the characteristic frequency of ***OctI_2_*** (**b**). XPS survey, N 1s, Pb 4f_7/2_ and I 3d_5/2_ spectra of glass/MAPbI_3_ (**c**) and glass/Oct(MA)_39_Pb_40_I_121_ (**d**) films before (as prepared) and after exposure to light for 1464 h (aged).

To identify the localization of the ***Oct*** dications within the perovskite films, scattering-type scanning infrared near-field optical microscopy (s-SNOM, IR-SNOM) was applied. This technique combines atomic force microscopy measurements and local infrared spectroscopy analysis with a lateral resolution of ca. 25–35 nm. Figure S5 shows the FTIR spectra of Cs_0.1_MA_0.15_FA_0.75_PbI_3_ and octenidine dihydroiodide powders recorded in the ATR (attenuated total reflection) mode, which were used to identify the ***OctI_2_*** characteristic frequency for mapping. For the perovskite absorber, a characteristic frequency of 1186 cm^−1^ was chosen. The topography and IR s-SNOM mapping images for the Oct(Cs_0.1_MA_0.15_FA_0.75_)_39_Pb_40_I_121_ film are presented in Figure 3. For the areas with higher concentration of the octenidine dihydroiodide a strong IR s-SNOM signal at the ***OctI_2_*** vibration frequency is observed (red areas), whereas low intensity (blue) areas are characterized by the decreased additive concentration. The obtained results revealed that the ***OctI_2_*** cations are distributed mainly between the perovskite grains and at their boundaries, whereas their content within the grains is much lower. Thus, IR s-SNOM data suggest that ***OctI_2_*** does not integrate within the MAPbI_3_ perovskite lattice but forms passivation shells at the grain boundaries most likely linking neighboring grains.

**Figure 4:**
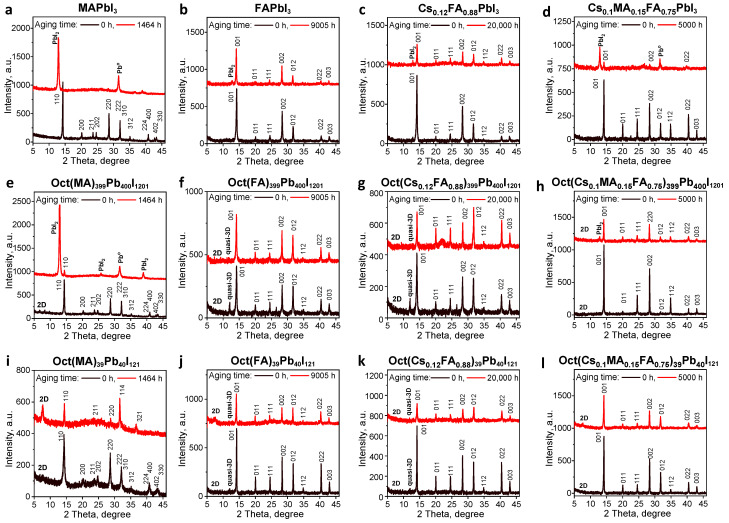
XRD patterns of the Oct(A)_n−1_Pb_n_I_3n+1_ films and the reference APbI_3_ samples before and after light soaking for 1464 h (A = MA) (**a**,**e**,**i**), 9000 h (A = FA) (**b**,**f**,**j**), 20,000 h (A = Cs_0.12_FA_0.88_) (**c**,**g**,**k**), and 5000 h (A = Cs_0.1_MA_0.15_FA_0.75_) (**d**,**h**,**l**).

Figure 4 shows the XRD patterns of the Oct(A)_n−1_Pb_n_I_3n+1_ films and the reference APbI_3_ samples before and after light exposure. According to these data, all the Oct(A)_n−1_Pb_n_I_3n+1_ films feature the main diffraction peaks corresponding to the 3D phase of APbI_3_. In particular, the pristine and modified absorbers based on MAPbI_3_ show distinct diffraction peaks at 14.2°, 20.2°, 23.6°, 24.6°, 26.8°, etc. characteristic of the tetragonal phase [86]. All the Oct(A)_n−1_Pb_n_I_3n+1_ (where A = FA, Cs_0.12_FA_0.88_ and A = Cs_0.1_MA_0.15_FA_0.75_) films exhibit the APbI_3_ perovskite cubic phase peaks corresponding to the (001), (011), (111), (002), (012), (022), and (003) diffraction planes, respectively [87]. However, virtually all Oct(A)_n−1_Pb_n_I_3n+1_ films reveal the additional peak at 7.6°, which can be attributed to the formation of 2D perovskite phase coexisting with the 3D perovskite phase [88,89]. The emergence of additional peaks at around 2θ = 12° and 26.5° for the material formulations with A = FA and Cs_0.12_FA_0.88_ is attributed to the formation of quasi-3D perovskites Oct(A)_n−1_Pb_n_I_3n+1_ (where n > 10) [90,91,92]. Figure S6 shows zoomed parts of the diffraction patterns of the Oct(A)_n−1_Pb_n_I_3n+1_ films depending on the loading of ***OctI_2_*** in comparison with the reference APbI_3_ samples. In the case of the modified systems based on MAPbI_3_ and Cs_0.12_FA_0.88_PbI_3_ at n = 400 and 40 we observed small shifts of the diffraction peaks by ~0.10 and 0.07°, respectively, towards larger 2θ angles. Such effect was not observed for other studied compositions. The slight shift of XRD peaks to higher angles indicates compression of the lattice, which might be caused by bulk cations located at the boundaries of perovskite grains and the lattice surface [93,94]. We believe that the 2D phases identified by XRD are localized mostly on the surface of the grains and at the grain boundaries as follows from the results of the IR s-SNOM mapping.

Some differences in the optical properties of the perovskite films induced by the ***Oct^2+^*** cation loading can be associated with the formation of quasi-3D perovskites featuring generally a slight blueshift of the absorption onset [92]. It is worth noting that low-intensity signals of unreacted PbI_2_ are observed at ~12.8° in the XRD patterns of the reference APbI_3_ perovskite (where A = MA, Cs_0.12_FA_0.88_) films and disappear after the addition of the ***OctI_2_*** additive, thus indicating its beneficial effect on suppressing the formation of inactive species during the perovskite formation.

To explore how the ***OctI_2_*** modification affects the intrinsic photostability of the perovskite films, the samples were exposed to a light soaking at 70 mW/cm^2^ and 38 ± 3 °C in an inert pure nitrogen atmosphere. It is clear that pristine MAPbI_3_ films undergo dramatic photobleaching (Figure 2a) due to the complete decomposition of the tetragonal perovskite phase as follows from the XRD data (Figure 4a). Lead iodide (PbI_2_) and metallic lead (Pb°) are the main degradation products as follows from the intense peaks at 12.6° and 31.6° (Figure 4a). On the contrary, the MAPbI_3_ films loaded with ***OctI_2_*** show a largely improved photostability as follows from the less pronounced changes in their optical properties and maintenance of the perovskite phase peaks in the XRD patterns of the OctMA_399_Pn_400_I_1201_ films (Figure 4e). Moreover, the increased ***OctI_2_*** loading in OctMA_39_Pb_40_I_121_ material formulation essentially blocks the light-induced decomposition of the MAPbI_3_ films as follows from a much weaker photobleaching with the preservation of the perovskite absorption band at 780 nm in their UV–Vis spectra, wherein XRD reveals no formation of PbI_2_ impurity even after aging for 1464 h (Figure 4i).

A substantial increase in the photostability of the MAPbI_3_ films loaded with ***OctI_2_*** was confirmed using X-ray photoelectron spectroscopy (XPS) (Figure 3c). The photochemical decomposition of MAPbI_3_ is accompanied by the loss of organic cations in the form of volatile organic species and the formation of PbI_2_ [95,96]. The XPS spectra confirmed this degradation pathway for unmodified MAPbI_3_: the N 1s band is completely quenched after 1464 h of light soaking showing a complete depletion of organic cations from the film surface, whereas the Pb 4f_7/2_ and, particularly, I 3d_5/2_ bands undergo significant high-energy shifts suggesting the formation of PbI_2_ and Pb° (Figure 3c). On the contrary, the OctMA_39_Pb_40_I_121_ film shows just a minor reduction in the intensity of the N 1s band after 1464 h of light soaking and much smaller shifts in the positions of the Pb 4f7/2 and I 3d5/2 bands (Figure 3d). These results prove unambiguously that the ***OctI_2_*** additive indeed effectively suppressed the photochemical degradation of MAPbI_3_.

In the case of the triple cation perovskite, the pristine films also undergo a considerable light-induced decomposition. The absorption spectra in Figure 2d show the appearance of strong light scattering due to severe recrystallization of the films and loss of their uniformity. Further, a dramatic photobleaching is observed after light soaking for 2411 h due to a near-complete decomposition of the cubic perovskite phase. According to the XRD data, lead iodide and metallic lead were the main degradation products after light soaking for 5000 h (Figure 4d). The engineered Oct(Cs_0.1_MA_0.15_FA_0.75_)_n−1_Pb_n_I_3n+1_ light absorbers demonstrated a spectacular resilience to the photoinduced degradation: a notably suppressed recrystallization of the films (Figure 2h,l) is observed, whereas XRD revealed only a slight admixture of lead iodide in the case of the material with n = 400 (Figure 4h). An increase in the ***OctI_2_*** loading (at n = 40) in the perovskite composition essentially blocks the light-induced decomposition of the absorber films since XRD reveals only the pure perovskite phase even after long exposure to light for 5000 h (Figure 4l).

In the case of the FAPbI_3_ and Cs_0.12_FA_0.88_PbI_3_ perovskites, the pristine films undergo a serious film recrystallization manifested in the appearance of severe scattering effects (Figure 2b,c). Interestingly, their phase composition remains almost unchanged and there is only a small amount of lead iodide appearing in the films after light soaking for 9000 and 20,000 h, respectively (Figure 4b,c). The deterioration of the perovskite film morphology is highly undesirable, because the carrier recombination, diffusion length, and carrier separation efficiency directly depend on it. The degradation of the photoactive layer morphology would lead to a sharp deterioration in the material charge-transport properties and, as a result, a drop in the photovoltaic device efficiency. The modification of the FAPbI_3_ absorber by ***OctI_2_*** results in a significant suppression of the light-scattering effects (Figure 2f,j), while Cs_0.12_FA_0.88_PbI_3_ perovskite shows almost complete blocking of the undesirable film recrystallization processes upon ***OctI_2_*** loading (Figure 2g,k).

The XRD patterns shown in Figure 4 reveal that the phase composition of the aged films is completely preserved and no signs of decomposition products appear after light exposure for 9000 h in the case of Oct(FA)_n−1_Pb_n_I_3n+1_ (Figure 4f,j) and 20,000 h for Oct(Cs_0.12_FA_0.88_)_n−1_Pb_n_I_3n+1_ (Figure 4g,k).

The observed substantial improvement in the material photostability can be attributed to the ability of divalent ***Oct^2+^*** cations to link the perovskite grains at the boundaries and decrease the density of surface defects though the chelating effect of pyridyl groups to the undercoordinated Pb^2+^ ions on the grain surface [27,97,98]. Passivation of defects on the perovskite grains and the grain boundaries represents a powerful tool to inhibit the photo-induced ion migration and the associated degradation mechanisms of the perovskite materials [10,24,99]. It is important to note that the obtained results are among the best values reported so far for photostability of non-encapsulated perovskite light-absorbing films (Table S1) [100,101].

**Figure 5:**
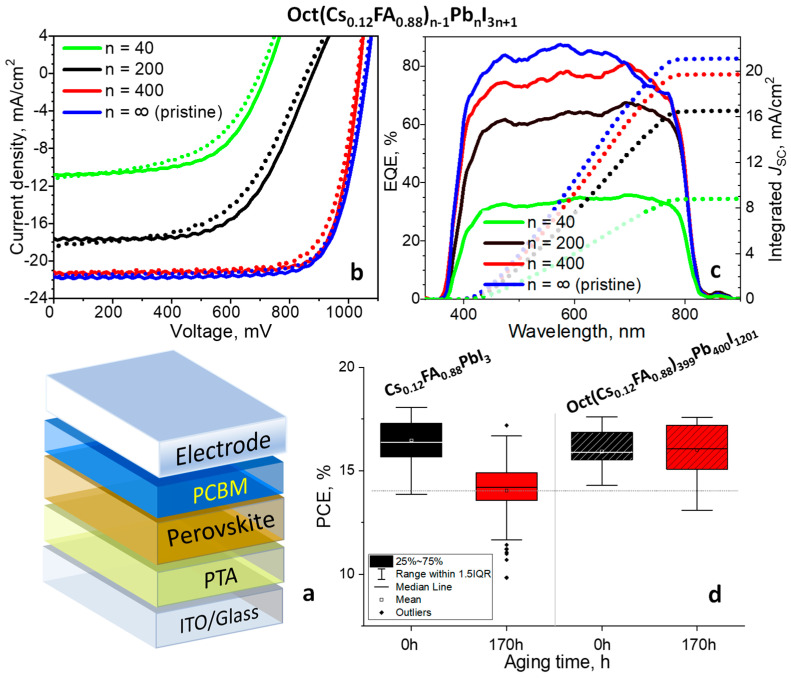
Device architecture (**a**), current–voltage characteristics (**b**), and EQE spectra (**b**) of the champion devices based on the pristine and modified perovskite films Oct(Cs_0.12_FA_0.88_)_n−1_Pb_n_I_3n+1_ with different ***OctI_2_*** contents (**c**). The power conversion efficiency (PCE) of the ITO/PTA/perovskite/PCBM/Al devices based on the pristine and modified Oct(Cs_0.12_FA_0.88_)_n−1_Pb_n_I_3n+1_ perovskite films before and after exposure to light (70 mW/cm^2^, T = 38 ± 3°C) for 170 h in inert atmosphere (**d**).

The effect of the ***OctI_2_*** cation on the photovoltaic performance of the OctA_n−1_Pb_n_I_3n+1_ (n = 40, 200, 400) absorber films was assessed for the Cs_0.12_FA_0.88_PbI_3_ perovskite system, which is highly promising due to its superior photostability [102]. The solar cells were fabricated with a planar p-i-n device architecture ITO/PTA/perovskite/PCBM/Ag (Figure 5a) in which poly[bis(4-phenyl)(4-methylphenyl)amine] (PTA) and phenyl-C_61_-butyric acid methyl ester (PC_61_BM) were used as hole-transport and electron-transport layer materials, respectively. The perovskite films were grown using the single-step spin-coating method. The device structure was completed by the evaporation of the top Ag electrodes (details are given in the Section 2).

Figure 5b shows the current density–voltage (J–V) characteristics for the champion cells fabricated using different perovskite compounds Oct(Cs_0.12_FA_0.88_)_n−1_Pb_n_I_3n+1_ (n = 40, 200, and 400) as well as the reference cells based on Cs_0.12_FA_0.88_PbI_3_, whereas the device photovoltaic parameters are summarized in Table S3 (Supplementary Materials). The short circuit current densities obtained in these measurements were reconfirmed by the integration of the EQE spectra against the standard AM1.5G solar irradiation spectrum as shown in Figure 5c.

The preliminary research without any additional optimization showed that the solar cell performance based on the Oct(Cs_0.12_FA_0.88_)_399_Pb_400_I_1201_ perovskite absorber was comparable to the reference devices featuring the power conversion efficiency (PCE) of 17.4% with the open circuit voltage (V_OC_) of 1035 mV, short circuit current density (*J*_SC_) of 21.3 mA·cm^−2^, and fill factor (FF) of 79.1% (Figure 5b, Table S3 (Supplementary Materials)). Meanwhile, the control device exhibited a maximum PCE of 17.7% with a *J*_SC_ of 21.7 mA·cm^−2^, V_OC_ of 1061 mV, and FF of 77.0%. Notably, the ***OctI_2_***-based cells demonstrated a slightly higher fill factor (FF) values as compared to the devices fabricated using non-domified perovskite films (Table S3 (Supplementary Materials)). This improvement may be associated with the suppressed carrier recombination due to a higher quality of absorber films with a lower density of structural defects and trap states. However, a further increase in ***OctI_2_*** loading in the perovskite layer leads to a severe decrease in all photovoltaic parameters presumably due to the deterioration of the charge collection due to the accumulation of 2D phase with anisotropic transport properties at the grain boundaries (Figure 5b, Table S3 (Supplementary Materials)). Unfortunately, low-dimensional A′A_n−1_Pb_n_I_3n+1_ perovskites with relatively low values of n generally exhibit high exciton binding energies and, hence, an inefficient generation and separation of charges that can limit their application in optoelectronic devices [103,104]. This problem might be overcome by the introduction of functional organic molecules, for instance, strong electron donors or acceptors [105,106].

The effect of the octenidine modification on the photostability of solar cells was explored using ITO/PTAA/absorber/PCBM stacks comprising Oct(Cs_0.12_FA_0.88_)_399_Pb_400_I_1201_ and Cs_0.12_FA_0.88_PbI_3_ compounds as photoactive layers. Half of the batch of the samples was exposed to light (LED, 70 mW/cm^2^, 38 ± 3 °C) under an inert nitrogen atmosphere for 170 h, whereas the other half was stored in the dark inside the glove box. After light exposure, the PCBM layer was refreshed to eliminate the influence of the electron-transporting layer degradation and then the metal top electrodes were deposited. Figure 3d and Figure S7 show that the ***OctI_2_*** modification notable enhances the photostability of solar cells. In particular, the devices based on pristine Cs_0.12_FA_0.88_PbI_3_ demonstrate a 15% reduction in efficiency after 170 h of light soaking as compared to the control devices kept in the dark. In contrast, the solar cells comprising Oct(Cs_0.12_FA_0.88_)_399_Pb_400_I_1201_ absorber showed practically the same performances for the devices exposed to light and stored in the dark (Figure 3d). We explain the increased stability of the octenidine-loaded devices by the healing of the defects such as undercoordinated Pb^2+^ cations on the surface of the grains and at the grain boundaries, which is translated to the superior PSCs operational stability as discussed below.

A particularly promising and simple approach is based on the surface modification of 3D perovskite films with bulk organic cations—inducing the formation of a thin shell of the 2D phase covering the surface of 3D perovskite grains and grain boundaries without causing any significant deterioration to the charge transport [107,108]. In addition, it is known that defects at the surface and grain boundaries in polycrystalline perovskite films serve as recombination sites leading to photogenerated carrier annihilation and degradation of absorber material [109]. Thus, following this approach, we investigated the possibility of increasing the stability of MAPbI_3_, FAPbI_3_, Cs_0.12_FA_0.88_PbI_3_, and Cs_0.1_MA_0.15_FA_0.85_PbI_3_ perovskites through surface modification with ***OctI_2_***. Technically, this modification is achieved by depositing a thin layer of the modifier on top of the grown perovskite films. Figure S8 (Supplementary Materials) shows the evolution of the UV−Vis absorption spectra for the surface-modified absorber films upon light exposure. The obtained results suggest that the stabilizing effect of surface modification could be even more impressive as compared to that achieved by introducing a modifying agent into the precursor solution for coating of perovskite films. The surface modification results in a slight red shift of the absorption band edge. Figure S9 (Supplementary Materials) shows the corresponding Tauc plots for the as-prepared reference and modified APbI_3_ samples. The observed bathochromic shift of the absorption onset can be explained by the accumulation of bulky organic cations mostly on the surface of the perovskite grains and at the grain boundaries [82].

Thus, the molecular additive engineering of perovskite absorber films using divalent ***Oct^2+^*** cations has been demonstrated to be an efficient technique to design material formulations with remarkably enhanced photostability, which is essential for reaching long operational lifetimes of PSCs.

## 4. Conclusions

Herein, we have introduced octenidine dihydroiodide ***OctI_2_*** as a highly promising molecular modifier for designing complex lead halides with spectacularly enhanced intrinsic photostability. Loading MAPbI_3_ and Cs_0.1_MA_0.15_FA_0.75_PbI_3_ perovskites with ***OctI_2_*** effectively suppresses film photobleaching effects, reduces photochemical depletion of organic cations and prevents the accumulation of aging products such as Pb° and PbI_2_. Most likely, divalent octenidinium cations bind to the surface of neighboring perovskite grains at the grain boundaries, passivate surface defects (such as underco-ordinated Pb^2+^ cations) and thus strongly stabilize the absorber material. In the case of FAPbI_3_ and Cs_0.12_FA_0.88_PbI_3_ perovskite formulations modified by incorporation of ***Oct^2+^*** cations, we observed a significant suppression or a complete blockade of the undesirable photoinduced perovskite recrystallization leading to the loss of film uniformity and the appearance of strong light-scattering effects. Furthermore, no signs of decomposition under white light exposure were observed for Oct(FA)_n−1_Pb_n_I_3n+1_ within 9000 h and Oct(Cs_0.12_FA_0.88_)_n−1_Pb_n_I_3n+1_ within 20,000 h, which are among the record lifetimes of perovskite films reported to date. Using the dication perovskite formulation as a model system, we showed that the optimally modified Oct(Cs_0.12_FA_0.88_)_399_Pb_400_I_1201_ perovskite layer delivers photovoltaic performances comparable to that of the reference devices based on the pristine Cs_0.12_FA_0.88_PbI_3_ absorber films.

The obtained results show that the proposed approach has great potential in the rational design of perovskite absorber materials with significantly enhanced photostability, and paves the way to a demonstration of efficient and durable perovskite solar cells that match industrially commercialized benchmarks.

## Data Availability

Data are contained within the article and Supplementary Materials.

## Supplementary Materials

The following supporting information can be downloaded at: https://www.mdpi.com/article/10.3390/ma17010129/s1, Figure S1: Absorption coefficients of the pristine and modified perovskite thin films as a function of the wavelength; Figure S2: Tauc plots for OctAn−1PbnI3n+1 films of various compositions used for Eg estimation (assuming direct bandgap); Figure S3: Surface morphology of the perovskite films: MAPbI_3_ (a), Oct(MA)_39_Pb_40_I_121_ (b), Cs_0.1_MA_0.15_FA_0.75_PbI_3_ (c), and Oct(Cs_0.1_MA_0.15_FA_0.75_)_39_Pb_40_I_121_ (d); Figure S4: Grain size distribution for the pristine MAPbI_3_ (a), Cs_0.1_MA_0.15_FA_0.75_PbI_3_ (c), modified Oct(MA)_39_Pb_40_I_121_ (b), and Oct(Cs_0.1_MA_0.15_FA_0.75_)_39_Pb_40_I_121_ (d) films; Figure S5: FTIR spectrum of the Cs_0.1_MA_0.15_FA_0.75_PbI_3_ and octenidine dihydroiodide powders; Figure S6: The zoomed parts of the diffraction patterns for the Oct(A)_n−1_Pb_n_I_3n+1_ films with different ***OctI_2_*** loadings compared to the reference APbI_3_ samples, where A = MA (a), FA (b), Cs_0.12_FA_0.88_ (c), and Cs_0.1_MA_0.15_FA_0.75_ (d); Figure S7: The open circuit voltage (V_OC_) (a), short circuit current density (J_SC_) (b), and fill factor values (FF) (c) of the ITO/PTA/perovskite/PCBM/Al devices based on Cs_0.12_FA_0.88_PbI_3_ and Oct(Cs_0.12_FA_0.88_)_399_Pb_400_I_1201_ absorber films with and w/o exposure to light for 170 h (70 mW/cm^2^, T = 38 ± 3 °C); Figure S8: The evolution of the UV–Vis spectra of the APbI_3_ films modified by the ***OctI_2_*** surface coating upon light exposure for A = MA (a), FA (b), Cs_0.12_FA_0.88_ (c), and Cs_0.1_MA_0.15_FA_0.75_ (d); Figure S9: Tauc plots for APbI_3_ films modified by the ***OctI_2_*** surface coating used for E*g* estimation (assuming direct bandgap) for A = MA (a), FA (b), Cs_0.12_FA_0.88_ (c), and Cs_0.1_MA_0.15_FA_0.75_ (d); Table S1: Overview of the relevant reported data on the perovskite solar cells based on the Dion–Jacobson absorber materials; Table S2: Experimental E*g* values and the absorption edge (in brackets) for the OctA_n−1_Pb_n_I_3n+1_ films of various compositions obtained from the corresponding Tauc plots; Table S3: Average and the best (in brackets) values of the device parameters based on Oct(Cs_0.12_FA_0.88_)_n−1_Pb_n_I_3n+1_ absorber layers with different ***OctI_2_*** contents.

## References

[1] NREL. https://www.nrel.gov/pv/cell-efficiency.html.

[2] Wang H., Dong Z., Liu H., Li W., Zhu L., Chen H. (2021). Roles of Organic Molecules in Inorganic CsPbX_3_ Perovskite Solar Cells. Adv. Energy Mater..

[3] Tu Y., Xu G., Yang X., Zhang Y., Li Z., Su R., Luo D., Yang W., Miao Y., Cai R. (2019). Mixed-cation perovskite solar cells in space. Sci. China Phys. Mech. Astron..

[4] Meng L., You J., Yang Y. (2018). Addressing the stability issue of perovskite solar cells for commercial applications. Nat. Commun..

[5] Akbulatov A.F., Luchkin S.Y., Frolova L.A., Dremova N.N., Gerasimov K.L., Zhidkov I.S., Anokhin D.V., Kurmaev E.Z., Stevenson K.J., Troshin P.A. (2017). Probing the Intrinsic Thermal and Photochemical Stability of Hybrid and Inorganic Lead Halide Perovskites. J. Phys. Chem. Lett..

[6] Mosconi E., Meggiolaro D., Snaith H.J., Stranks S.D., De Angelis F. (2016). Light-Induced Annihilation of Frenkel Defects in Organo-Lead Halide Perovskites. Energy Environ. Sci..

[7] Misra R.K., Aharon S., Li B., Mogilyansky D., Visoly-Fisher I., Etgar L., Katz E.A. (2015). Temperature- and Component-Dependent Degradation of Perovskite Photovoltaic Materials under Concentrated Sunlight. J. Phys Chem. Lett..

[8] Conings B., Drijkoningen J., Gauquelin N., Babayigit A., D’Haen J., D’Olieslaeger L., Ethirajan A., Verbeeck J., Manca J., Mosconi E. (2015). Intrinsic Thermal Instability of Methylammonium Lead Trihalide Perovskite. Adv. Energy Mater..

[9] Leguy A.M.A., Hu Y., Campoy-Quiles M., Alonso M.I., Weber O.J., Azarhoosh P., van Schilfgaarde M., Weller M.T., Bein T., Nelson J. (2015). Reversible Hydration of CH_3_NH_3_PbI_3_ in Films, Single Crystals, and Solar Cells. Chem. Mater..

[10] Miah M.H., Rahman M.B., Nur-E-Alam M., Das N., Soin N.B., Hatta S.F.W.M., Islam M.A. (2023). Understanding the Degradation Factors, Mechanism and Initiatives for Highly Efficient Perovskite Solar Cells. ChemNanoMat.

[11] Yuan M., Quan L.N., Comin R., Walters G., Sabatini R., Voznyy O., Hoogland S., Zhao Y., Beauregard E.M., Kanjanaboos P. (2016). Perovskite Energy Funnels for Efficient Light-Emitting Diodes. Nat. Nanotechnol..

[12] Zhou N., Shen Y., Li L., Tan S., Liu N., Zheng G., Chen Q., Zhou H. (2018). Exploration of Crystallization Kinetics in Quasi Two-Dimensional Perovskite and High Performance Solar Cells. J. Am. Chem. Soc..

[13] Ye Q., Ma F., Zhao Y., Yu S., Chu Z., Gao P., Zhang X., You J. (2020). Stabilizing γ-CsPbI _3_ Perovskite via Phenylethylammonium for Efficient Solar Cells with Open-Circuit Voltage over 1.3 V. Small.

[14] Zhou T., Lai H., Liu T., Lu D., Wan X., Zhang X., Liu Y., Chen Y. (2019). Highly Efficient and Stable Solar Cells Based on Crystalline Oriented 2D/3D Hybrid Perovskite. Adv. Mater..

[15] Yella A., Heiniger L.-P., Gao P., Nazeeruddin M.K., Grätzel M. (2014). Nanocrystalline Rutile Electron Extraction Layer Enables Low-Temperature Solution Processed Perovskite Photovoltaics with 13.7% Efficiency. Nano Lett..

[16] Liang C., Gu H., Xia Y., Wang Z., Liu X., Xia J., Zuo S., Hu Y., Gao X., Hui W. (2020). Two-Dimensional Ruddlesden–Popper Layered Perovskite Solar Cells Based on Phase-Pure Thin Films. Nat. Energy.

[17] Wu W.-Q., Rudd P.N., Ni Z., Van Brackle C.H., Wei H., Wang Q., Ecker B.R., Gao Y., Huang J. (2020). Reducing Surface Halide Deficiency for Efficient and Stable Iodide-Based Perovskite Solar Cells. J. Am. Chem. Soc..

[18] Tsai H., Nie W., Blancon J.-C., Stoumpos C.C., Asadpour R., Harutyunyan B., Neukirch A.J., Verduzco R., Crochet J.J., Tretiak S. (2016). High-Efficiency Two-Dimensional Ruddlesden–Popper Perovskite Solar Cells. Nature.

[19] Straus D.B., Kagan C.R. (2018). Electrons, Excitons, and Phonons in Two-Dimensional Hybrid Perovskites: Connecting Structural, Optical, and Electronic Properties. J. Phys. Chem. Lett..

[20] Huang Z., Proppe A.H., Tan H., Saidaminov M.I., Tan F., Mei A., Tan C.-S., Wei M., Hou Y., Han H. (2019). Suppressed Ion Migration in Reduced-Dimensional Perovskites Improves Operating Stability. ACS Energy Lett..

[21] Lu J., Jiang L., Li W., Li F., Pai N.K., Scully A.D., Tsai C., Bach U., Simonov A.N., Cheng Y. (2017). Diammonium and Monoammonium Mixed-Organic-Cation Perovskites for High Performance Solar Cells with Improved Stability. Adv. Energy Mater..

[22] He Q., Worku M., Xu L., Zhou C., Lin H., Robb A.J., Hanson K., Xin Y., Ma B. (2020). Facile Formation of 2D–3D Heterojunctions on Perovskite Thin Film Surfaces for Efficient Solar Cells. ACS Appl. Mater. Interfaces.

[23] Jiang X., Zhang J., Ahmad S., Tu D., Liu X., Jia G., Guo X., Li C. (2020). Dion-Jacobson 2D-3D Perovskite Solar Cells with Improved Efficiency and Stability. Nano Energy.

[24] Niu T., Ren H., Wu B., Xia Y., Xie X., Yang Y., Gao X., Chen Y., Huang W. (2019). Reduced-Dimensional Perovskite Enabled by Organic Diamine for Efficient Photovoltaics. J. Phys. Chem. Lett..

[25] Wang J., Lin D., Chen Y., Luo S., Ke L., Ren X., Cui S., Zhang L., Li Z., Meng K. (2020). Suppressing the Excessive Solvated Phase for Dion–Jacobson Perovskites with Improved Crystallinity and Vertical Orientation. Sol. RRL.

[26] Zhang F., Lu H., Tong J., Berry J.J., Beard M.C., Zhu K. (2020). Advances in Two-Dimensional Organic–inorganic Hybrid Perovskites. Energy Environ. Sci..

[27] Paritmongkol W., Dahod N.S., Stollmann A., Mao N., Settens C., Zheng S.-L., Tisdale W.A. (2019). Synthetic Variation and Structural Trends in Layered Two-Dimensional Alkylammonium Lead Halide Perovskites. Chem. Mater..

[28] Sourisseau S., Louvain N., Bi W., Mercier N., Rondeau D., Boucher F., Buzaré J.-Y., Legein C. (2007). Reduced Band Gap Hybrid Perovskites Resulting from Combined Hydrogen and Halogen Bonding at the Organic−Inorganic Interface. Chem. Mater..

[29] Zheng Y., Niu T., Qiu J., Chao L., Li B., Yang Y., Li Q., Lin C., Gao X., Zhang C. (2019). Oriented and Uniform Distribution of Dion–Jacobson Phase Perovskites Controlled by Quantum Well Barrier Thickness. Sol. RRL.

[30] Mao L., Ke W., Pedesseau L., Wu Y., Katan C., Even J., Wasielewski M.R., Stoumpos C.C., Kanatzidis M.G. (2018). Hybrid Dion–Jacobson 2D Lead Iodide Perovskites. J. Am. Chem. Soc..

[31] Ahmad S., Fu P., Yu S., Yang Q., Liu X., Wang X., Wang X., Guo X., Li C. (2019). Dion-Jacobson Phase 2D Layered Perovskites for Solar Cells with Ultrahigh Stability. Joule.

[32] Ma C., Shen D., Ng T., Lo M., Lee C. (2018). 2D Perovskites with Short Interlayer Distance for High-Performance Solar Cell Application. Adv. Mater..

[33] Huang P., Kazim S., Wang M., Ahmad S. (2019). Toward Phase Stability: Dion–Jacobson Layered Perovskite for Solar Cells. ACS Energy Lett..

[34] Quan L.N., Yuan M., Comin R., Voznyy O., Beauregard E.M., Hoogland S., Buin A., Kirmani A.R., Zhao K., Amassian A. (2016). Ligand-Stabilized Reduced-Dimensionality Perovskites. J. Am. Chem. Soc..

[35] Niu T., Lu J., Tang M.-C., Barrit D., Smilgies D.-M., Yang Z., Li J., Fan Y., Luo T., McCulloch I. (2018). High Performance Ambient-Air-Stable FAPbI_3_ Perovskite Solar Cells with Molecule-Passivated Ruddlesden–Popper/3D Heterostructured Film. Energy Environ. Sci..

[36] Wang H., Qin Z., Xie J., Zhao S., Liu K., Guo X., Li G., Lu X., Yan K., Xu J. (2020). Efficient Slantwise Aligned Dion–Jacobson Phase Perovskite Solar Cells Based on Trans-1,4-Cyclohexanediamine. Small.

[37] Cheng L., Liu Z., Li S., Zhai Y., Wang X., Qiao Z., Xu Q., Meng K., Zhu Z., Chen G. (2021). Highly Thermostable and Efficient Formamidinium-Based Low-Dimensional Perovskite Solar Cells. Angew. Chem. Int. Ed..

[38] Li Y., Milić J.V., Ummadisingu A., Seo J.-Y., Im J.-H., Kim H.-S., Liu Y., Dar M.I., Zakeeruddin S.M., Wang P. (2019). Bifunctional Organic Spacers for Formamidinium-Based Hybrid Dion–Jacobson Two-Dimensional Perovskite Solar Cells. Nano Lett..

[39] Cohen B.-E., Li Y., Meng Q., Etgar L. (2019). Dion–Jacobson Two-Dimensional Perovskite Solar Cells Based on Benzene Dimethanammonium Cation. Nano Lett..

[40] Su P., Bai L., Bi H., Liu B., Chen S., Lee D., Yang H., Chen C., Zang Z., Chen J. (2021). Interfacial Gradient Energy Band Alignment Modulation via Ion Exchange Reaction toward Efficient and Stable Methylammonium-Free Dion-Jacobson Quasi-2D Perovskite Solar Cells. J. Power Sources.

[41] Fang Z., Shang M., Zheng Y., Zhang T., Du Z., Wang G., Duan X., Chou K.-C., Lin C.-H., Yang W. (2020). Organic Intercalation Engineering of Quasi-2D Dion–Jacobson A-CsPbI_3_ Perovskites. Mater. Horiz..

[42] Safdari M., Phuyal D., Philippe B., Svensson P.H., Butorin S.M., Kvashnina K.O., Rensmo H., Kloo L., Gardner J.M. (2017). Impact of Synthetic Routes on the Structural and Physical Properties of Butyl-1,4-Diammonium Lead Iodide Semiconductors. J. Mater. Chem. A.

[43] Hou J., Deng F., Wu Q., Yang L., Wu J., Li X., Zheng Y.-Z., Li N., Ding H., Tao X. (2020). Ethylenediamine Chlorides Additive Assisting Formation of High-Quality Formamidinium-Caesium Perovskite Film with Low Trap Density for Efficient Solar Cells. J. Power Sources.

[44] Zhang T., Dar M.I., Li G., Xu F., Guo N., Grätzel M., Zhao Y. (2017). Bication Lead Iodide 2D Perovskite Component to Stabilize Inorganic A-CsPbI_3_ Perovskite Phase for High-Efficiency Solar Cells. Sci. Adv..

[45] Jokar E., Chien C.-H., Fathi A., Rameez M., Chang Y.-H., Diau E.W.-G. (2018). Slow Surface Passivation and Crystal Relaxation with Additives to Improve Device Performance and Durability for Tin-Based Perovskite Solar Cells. Energy Environ. Sci..

[46] Zheng H., Liu G., Zhu L., Ye J., Zhang X., Alsaedi A., Hayat T., Pan X., Dai S. (2018). The Effect of Hydrophobicity of Ammonium Salts on Stability of Quasi-2D Perovskite Materials in Moist Condition. Adv. Energy Mater..

[47] Wang Y., Liu S., Zeng Q., Wang R., Qin W., Cao H., Yang L., Li L., Yin S., Zhang F. (2018). Enhanced Performance and Stability of Inverted Planar Perovskite Solar Cells by Incorporating 1,6-Diaminohexane Dihydrochloride Additive. Sol. Energy Mater. Sol. Cells.

[48] Luo W., Wu C., Wang D., Zhang Y., Zhang Z., Qi X., Zhu N., Guo X., Qu B., Xiao L. (2019). Efficient and Stable Perovskite Solar Cell with High Open-Circuit Voltage by Dimensional Interface Modification. ACS Appl. Mater. Interfaces.

[49] Zhao T., Chueh C.-C., Chen Q., Rajagopal A., Jen A.K.-Y. (2016). Defect Passivation of Organic–Inorganic Hybrid Perovskites by Diammonium Iodide toward High-Performance Photovoltaic Devices. ACS Energy Lett..

[50] Li P., Zhang Y., Liang C., Xing G., Liu X., Li F., Liu X., Hu X., Shao G., Song Y. (2018). Phase Pure 2D Perovskite for High-Performance 2D–3D Heterostructured Perovskite Solar Cells. Adv. Mater..

[51] Xin D., Tie S., Yuan R., Zheng X., Zhu J., Zhang W.-H. (2019). Defect Passivation in Hybrid Perovskite Solar Cells by Tailoring the Electron Density Distribution in Passivation Molecules. ACS Appl. Mater. Interfaces.

[52] Chen J., Kim S.-G., Ren X., Jung H.S., Park N.-G. (2019). Effect of Bidentate and Tridentate Additives on the Photovoltaic Performance and Stability of Perovskite Solar Cells. J. Mater. Chem. A.

[53] Lee M.-S., Sarwar S., Park S., Asmat U., Thuy D.T., Han C., Ahn S., Jeong I., Hong S. (2020). Efficient Defect Passivation of Perovskite Solar Cells via Stitching of an Organic Bidentate Molecule. Sustain. Energy Fuels.

[54] Salado M., Jodlowski A.D., Roldan-Carmona C., de Miguel G., Kazim S., Nazeeruddin M.K., Ahmad S. (2018). Surface Passivation of Perovskite Layers Using Heterocyclic Halides: Improved Photovoltaic Properties and Intrinsic Stability. Nano Energy.

[55] Shen D., Wu W., Li Y., Abate A., Wei M. (2020). 2-Methylimidazole as an Interlayer for the Enhancement of the Open-Circuit Voltage in Perovskite Solar Cells. J. Power Sources.

[56] Ke W., Mao L., Stoumpos C.C., Hoffman J., Spanopoulos I., Mohite A.D., Kanatzidis M.G. (2019). Compositional and Solvent Engineering in Dion–Jacobson 2D Perovskites Boosts Solar Cell Efficiency and Stability. Adv. Energy Mater..

[57] Wu H., Lian X., Tian S., Zhang Y., Qin M., Zhang Y., Wang F., Lu X., Wu G., Chen H. (2020). Additive-Assisted Hot-Casting Free Fabrication of Dion–Jacobson 2D Perovskite Solar Cell with Efficiency Beyond 16%. Solar RRL.

[58] Li X., Ke W., Traoré B., Guo P., Hadar I., Kepenekian M., Even J., Katan C., Stoumpos C.C., Schaller R.D. (2019). Two-Dimensional Dion–Jacobson Hybrid Lead Iodide Perovskites with Aromatic Diammonium Cations. J. Am. Chem. Soc..

[59] Lu D., Lv G., Xu Z., Dong Y., Ji X., Liu Y. (2020). Thiophene-Based Two-Dimensional Dion–Jacobson Perovskite Solar Cells with over 15% Efficiency. J. Am. Chem. Soc..

[60] Zhao W., Dong Q., Zhang J., Wang S., Chen M., Zhao C., Hu M., Jin S., Padture N.P., Shi Y. (2020). Asymmetric Alkyl Diamine Based Dion–Jacobson Low-Dimensional Perovskite Solar Cells with Efficiency Exceeding 15%. J. Mater. Chem. A.

[61] Safdari M., Svensson P.H., Hoang M.T., Oh I., Kloo L., Gardner J.M. (2016). Layered 2D Alkyldiammonium Lead Iodide Perovskites: Synthesis, Characterization, and Use in Solar Cells. J. Mater. Chem. A.

[62] Li F., Zhang J., Jo S., Qin M., Li Z., Liu T., Lu X., Zhu Z., Jen A.K.-Y. (2020). Vertical Orientated Dion–Jacobson Quasi-2D Perovskite Film with Improved Photovoltaic Performance and Stability. Small Methods.

[63] Zhao X., Liu T., Kaplan A.B., Yao C., Loo Y.-L. (2020). Accessing Highly Oriented Two-Dimensional Perovskite Films via Solvent-Vapor Annealing for Efficient and Stable Solar Cells. Nano Lett..

[64] Li P., Liu X., Zhang Y., Liang C., Chen G., Li F., Su M., Xing G., Tao X., Song Y. (2020). Low-Dimensional Dion–Jacobson-Phase Lead-Free Perovskites for High-Performance Photovoltaics with Improved Stability. Angew. Chem. Int. Ed..

[65] Wang H., Chan C.C.S., Chu M., Xie J., Zhao S., Guo X., Miao Q., Wong K.S., Yan K., Xu J. (2020). Interlayer Cross-Linked 2D Perovskite Solar Cell with Uniform Phase Distribution and Increased Exciton Coupling. Solar RRL.

[66] Jin L., Ren N., Wang P., Li R., Xue Q., Huang F., Zhang X., Zhao Y., Zhang X. (2023). Secondary Anti-Solvent Treatment for Efficient 2D Dion–Jacobson Perovskite Solar Cells. Small.

[67] Lv G., Li L., Lu D., Xu Z., Dong Y., Li Q., Chang Z., Yin W.-J., Liu Y. (2021). Multiple-Noncovalent-Interaction-Stabilized Layered Dion–Jacobson Perovskite for Efficient Solar Cells. Nano Lett..

[68] Xu Z., Lu D., Dong X., Chen M., Fu Q., Liu Y. (2021). Highly Efficient and Stable Dion−Jacobson Perovskite Solar Cells Enabled by Extended π-Conjugation of Organic Spacer. Adv. Mater..

[69] Chen Z., Liu M., Li Z., Shi T., Yang Y., Yip H.-L., Cao Y. (2018). Stable Sn/Pb-Based Perovskite Solar Cells with a Coherent 2D/3D Interface. iScience.

[70] Yang G., Ren Z., Liu K., Qin M., Deng W., Zhang H., Wang H., Liang J., Ye F., Liang Q. (2021). Stable and Low-Photovoltage-Loss Perovskite Solar Cells by Multifunctional Passivation. Nat. Photonics.

[71] Ma K., Atapattu H.R., Zhao Q., Gao Y., Finkenauer B.P., Wang K., Chen K., Park S.M., Coffey A.H., Zhu C. (2021). Multifunctional Conjugated Ligand Engineering for Stable and Efficient Perovskite Solar Cells. Adv. Mater..

[72] Kim D.H., Lee C.M., Islam A., Choi D.H., Jeong G., Kim T.W., Cho H.W., Kim Y.B., Shah S.H.U., Park M.J. (2022). Efficient Photon Extraction in Top-Emission Organic Light-Emitting Devices Based on Ampicillin Microstructures. Adv. Mater..

[73] Ali A., Jiang W., Choi Y., Jeon E., Chae H. (2022). Enhanced Charge Balance with Antibiotics in Both Electron and Hole Transport Layers of InP/ZnSexS1−x/ZnS-Based Quantum Dot Light-Emitting Diodes. J. Alloys Compd..

[74] Mazumdar S., Zhao Y., Zhang X. (2021). Stability of Perovskite Solar Cells: Degradation Mechanisms and Remedies. Front. Electron..

[75] Yakuhenko I.K., Pozdeeva N.N., Mumyatova V.A., Terentev A.A., Gadomskij S.V. (2019). Metod of Producing Octenidine Isomer and Use Thereof a Antibacterial and Antifungal Agent of Wide Action Spectrum. Russian Federation Patent.

[76] Harke H.-P. (2000). Disinfectants. Ullmann’s Encyclopedia of Industrial Chemistry.

[77] Gastmeier P., Kämpf K.-P., Behnke M., Geffers C., Schwab F. (2016). An Observational Study of the Universal Use of Octenidine to Decrease Nosocomial Bloodstream Infections and MDR Organisms. J. Antimicrob. Chemother..

[78] Al-Doori Z., Goroncy-Bermes P., Gemmell C.G., Morrison D. (2007). Low-Level Exposure of MRSA to Octenidine Dihydrochloride Does Not Select for Resistance. J. Antimicrob. Chemother..

[79] Ozerova V.V., Zhidkov I.S., Boldyreva A., Dremova N.N., Emelianov N.A., Shilov G.V., Frolova L.A., Kurmaev E.Z., Sukhorukov A.Y., Aldoshin S.M. (2021). Spectacular Enhancement of the Thermal and Photochemical Stability of MAPbI3 Perovskite Films Using Functionalized Tetraazaadamantane as a Molecular Modifier. Energies.

[80] Adonin S.A., Frolova L.A., Sokolov M.N., Shilov G.V., Korchagin D.V., Fedin V.P., Aldoshin S.M., Stevenson K.J., Troshin P.A. (2018). Antimony (V) Complex Halides: Lead-Free Perovskite-Like Materials for Hybrid Solar Cells. Adv. Energy Mater..

[81] Zhi C., Li Z., Wei B. (2021). Recent Progress in Stabilizing Perovskite Solar Cells through Two-Dimensional Modification. APL Mater..

[82] Ma J., Li W., Le N.T., Díaz-Real J.A., Body M., Legein C., Światowska J., Demortière A., Borkiewicz O.J., Konstantinova E.A. (2019). Red-Shifted Absorptions of Cation-Defective and Surface-Functionalized Anatase with Enhanced Photoelectrochemical Properties. ACS Omega.

[83] Yuan Z., You W., Jia J., Zhang L. (1998). Optical Absorption Red Shift of Capped ZnFe_2_O_4_ Nanoparticle. Chin. Phys. Lett..

[84] Zhu L., Cao H., Xue C., Zhang H., Qin M., Wang J., Wen K., Fu Z., Jiang T., Xu L. (2021). Unveiling the Additive-Assisted Oriented Growth of Perovskite Crystallite for High Performance Light-Emitting Diodes. Nat. Commun..

[85] Aiello F., Masi S. (2021). The Contribution of NMR Spectroscopy in Understanding Perovskite Stabilization Phenomena. Nanomaterials.

[86] Shao Y., Xiao Z., Bi C., Yuan Y., Huang J. (2014). Origin and Elimination of Photocurrent Hysteresis by Fullerene Passivation in CH_3_NH_3_PbI_3_ Planar Heterojunction Solar Cells. Nat. Commun..

[87] Murugadoss G., Thangamuthu R., Rajesh Kumar M. (2018). Formamidinium Lead Iodide Perovskite: Structure, Shape and Optical Tuning via Hydrothermal Method. Mater. Lett..

[88] Bidikoudi M., Simal C., Dracopoulos V., Stathatos E. (2021). Exploring the Effect of Ammonium Iodide Salts Employed in Multication Perovskite Solar Cells with a Carbon Electrode. Molecules.

[89] Poli I., Eslava S., Cameron P. (2017). Tetrabutylammonium Cations for Moisture-Resistant and Semitransparent Perovskite Solar Cells. J. Mater. Chem. A.

[90] Stoumpos C.C., Soe C.M.M., Tsai H., Nie W., Blancon J.-C., Cao D.H., Liu F., Traoré B., Katan C., Even J. (2017). High Members of the 2D Ruddlesden-Popper Halide Perovskites: Synthesis, Optical Properties, and Solar Cells of (CH_3_(CH_2_)_3_NH_3_)_2_(CH_3_NH_3_)_4_Pb_5_I_16_. Chem.

[91] Yan Y., Yang Y., Liang M., Abdellah M., Pullerits T., Zheng K., Liang Z. (2021). Implementing an Intermittent Spin-Coating Strategy to Enable Bottom-up Crystallization in Layered Halide Perovskites. Nat. Commun..

[92] Lee J.-W., Dai Z., Han T.-H., Choi C., Chang S.-Y., Lee S.-J., De Marco N., Zhao H., Sun P., Huang Y. (2018). 2D Perovskite Stabilized Phase-Pure Formamidinium Perovskite Solar Cells. Nat. Commun..

[93] Smith I.C., Hoke E.T., Solis-Ibarra D., McGehee M.D., Karunadasa H.I. (2014). A Layered Hybrid Perovskite Solar-Cell Absorber with Enhanced Moisture Stability. Angew. Chem. Int. Ed. Engl..

[94] Hervoches C.H., Lightfoot P. (1999). A Variable-Temperature Powder Neutron Diffraction Study of Ferroelectric Bi_4_Ti_3_O_12_. Chem. Mater..

[95] Juarez-Perez E.J., Ono L.K., Maeda M., Jiang Y., Hawash Z., Qi Y. (2018). Photodecomposition and Thermal Decomposition in Methylammonium Halide Lead Perovskites and Inferred Design Principles to Increase Photovoltaic Device Stability. J. Mater. Chem. A.

[96] Akbulatov A.F., Frolova L.A., Dremova N.N., Zhidkov I., Martynenko V.M., Tsarev S.A., Luchkin S.Y., Kurmaev E.Z., Aldoshin S.M., Stevenson K.J. (2020). Light or Heat: What Is Killing Lead Halide Perovskites under Solar Cell Operation Conditions?. J. Phys. Chem. Lett..

[97] Zhang J., Wang L., Jiang C., Cheng B., Chen T., Yu J. (2021). CsPbBr_3_ Nanocrystal Induced Bilateral Interface Modification for Efficient Planar Perovskite Solar Cells. Adv. Sci..

[98] Cao J., Lv X., Zhang P., Chuong T.T., Wu B., Feng X., Shan C., Liu J., Tang Y. (2018). Plant Sunscreen and Co(II)/(III) Porphyrins for UV-Resistant and Thermally Stable Perovskite Solar Cells: From Natural to Artificial. Adv. Mater..

[99] Chen X., Xia Y., Huang Q., Li Z., Mei A., Hu Y., Wang T., Cheacharoen R., Rong Y., Han H. (2021). Tailoring the Dimensionality of Hybrid Perovskites in Mesoporous Carbon Electrodes for Type-II Band Alignment and Enhanced Performance of Printable Hole-Conductor-Free Perovskite Solar Cells. Adv. Energy Mater..

[100] Lin T., Dai T., Li X. (2023). 2D/3D Perovskite: A Step toward Commercialization of Perovskite Solar Cells. Sol. RRL.

[101] Li X., Zhang P., Li S., Wasnik P., Ren J., Jiang Q., Xu B.B., Murugadoss V. (2023). Mixed Perovskites (2D/3D)-Based Solar Cells: A Review on Crystallization and Surface Modification for Enhanced Efficiency and Stability. Adv. Compos. Hybrid Mater..

[102] Akbulatov A.F., Ustinova M.I., Gutsev L., Tsarev S.A., Dremova N.N., Zhidkov I., Luchkin S.Y., Ramachandran B.R., Frolova L., Kurmaev E.Z. (2021). When Iodide Meets Bromide: Halide Mixing Facilitates the Light-Induced Decomposition of Perovskite Absorber Films. Nano Energy.

[103] Tanaka K., Kondo T. (2003). Bandgap and Exciton Binding Energies in Lead-Iodide-Based Natural Quantum-Well Crystals. Sci. Technol. Adv. Mater..

[104] Muljarov E.A., Tikhodeev S.G., Gippius N.A., Ishihara T. (1995). Excitons in Self-Organized Semiconductor/insulator Superlattices: PbI-Based Perovskite Compounds. Phys. Rev. B.

[105] Maheshwari S., Savenije T.J., Renaud N., Grozema F.C. (2018). Computational Design of Two-Dimensional Perovskites with Functional Organic Cations. J. Phys. Chem. C.

[106] Gélvez-Rueda M.C., Fridriksson M.B., Dubey R.K., Jager W.F., van der Stam W., Grozema F.C. (2020). Overcoming the Exciton Binding Energy in Two-Dimensional Perovskite Nanoplatelets by Attachment of Conjugated Organic Chromophores. Nat. Commun..

[107] Grancini G., Roldán-Carmona C., Zimmermann I., Mosconi E., Lee X., Martineau D., Narbey S., Oswald F., De Angelis F., Graetzel M. (2017). One-Year Stable Perovskite Solar Cells by 2D/3D Interface Engineering. Nat. Commun..

[108] Shen X., Liu C., Wen F., Zhou X., Liao J., Li H. (2023). Localization Control of 2D/3D Perovskite Heterostructures at Grain Boundaries by Amine-Vapor-Induced Dimensionality Reduction. J. Alloys Compd..

[109] Zhang Z., Gao Y., Li Z., Qiao L., Xiong Q., Deng L., Zhang Z., Long R., Zhou Q., Du Y. (2021). Marked Passivation Effect of Naphthalene-1,8-Dicarboximides in High-Performance Perovskite Solar Cells. Adv. Mater..

