# Supplementary material for: Enhancing Photostability of Complex Lead Halides through Modification with Antibacterial Drug Octenidine
Source: Materials (Basel). 2023 Dec 26;17(1):129. doi: 10.3390/ma17010129 (PMC10780031; doi:10.3390/ma17010129)
Supplement: Supplementary file 1 [file materials-17-00129-s001.zip › materials-2738106-supplementary.pdf]

Supplementary Materials

Enhancing photostability of complex lead halides through modification with antibacterial drug octenidine

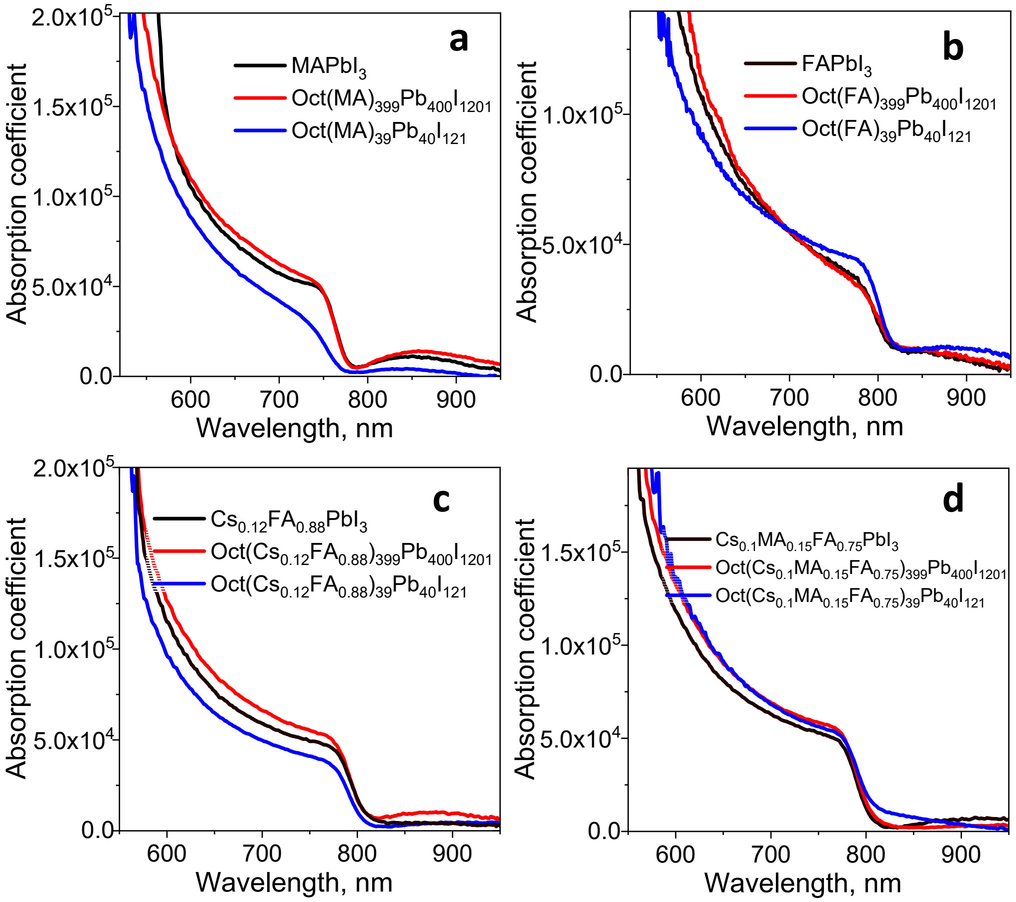

Figure S1. Absorption coefficients of the pristine and modified perovskite thin films as a function of the wavelength.

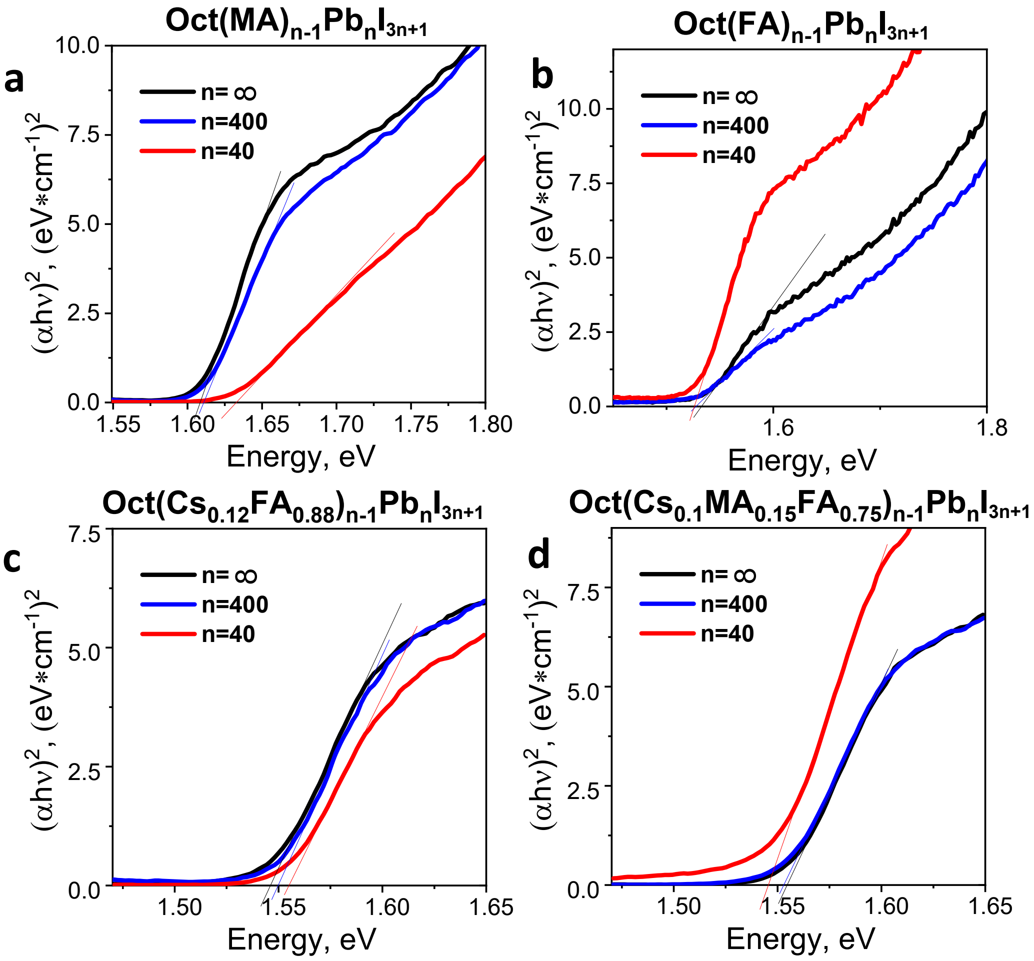

Figure S2. Tauc plots for the $OctA_{n-1}Pb_nI_{3n+1}$ films of various compositions used for E_g estimation (assuming direct bandgap).

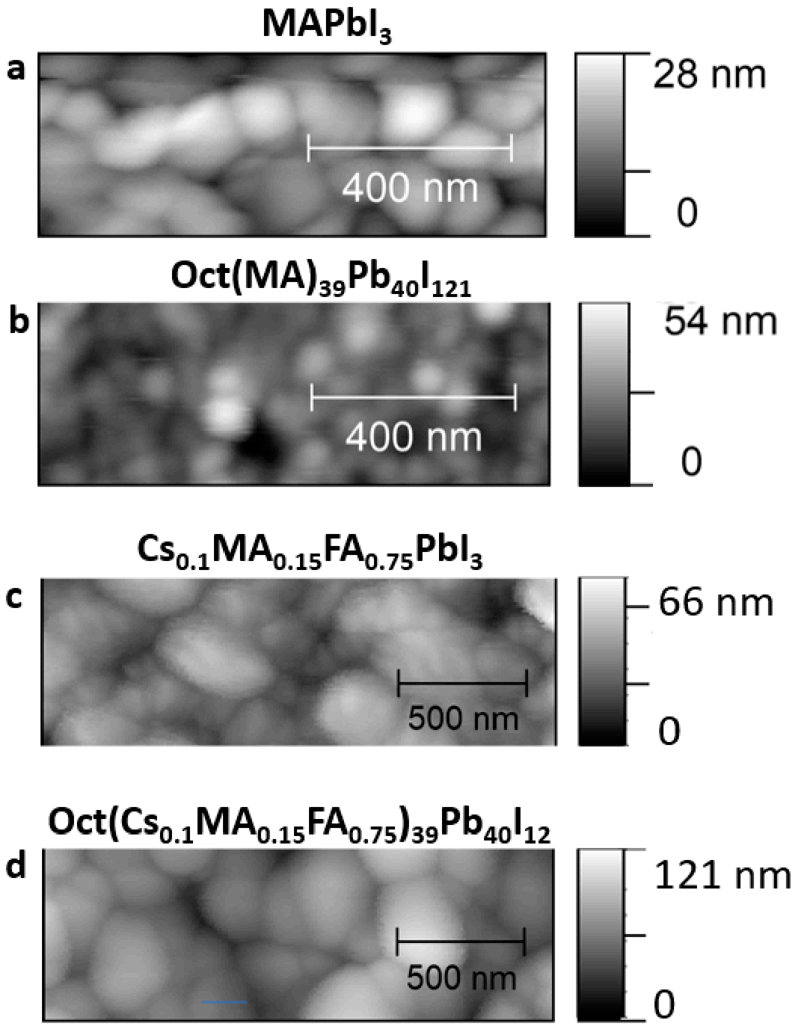

Figure S3. Surface morphology of the perovskite films: $MAPbI_3$ (a), $Oct(MA)_{39}Pb_{40}I_{121}$ (b), $Cs_{0.1}MA_{0.15}FA_{0.75}PbI_3$ (c), and $Oct(Cs_{0.1}MA_{0.15}FA_{0.75})_{39}Pb_{40}I_{121}$ (d).

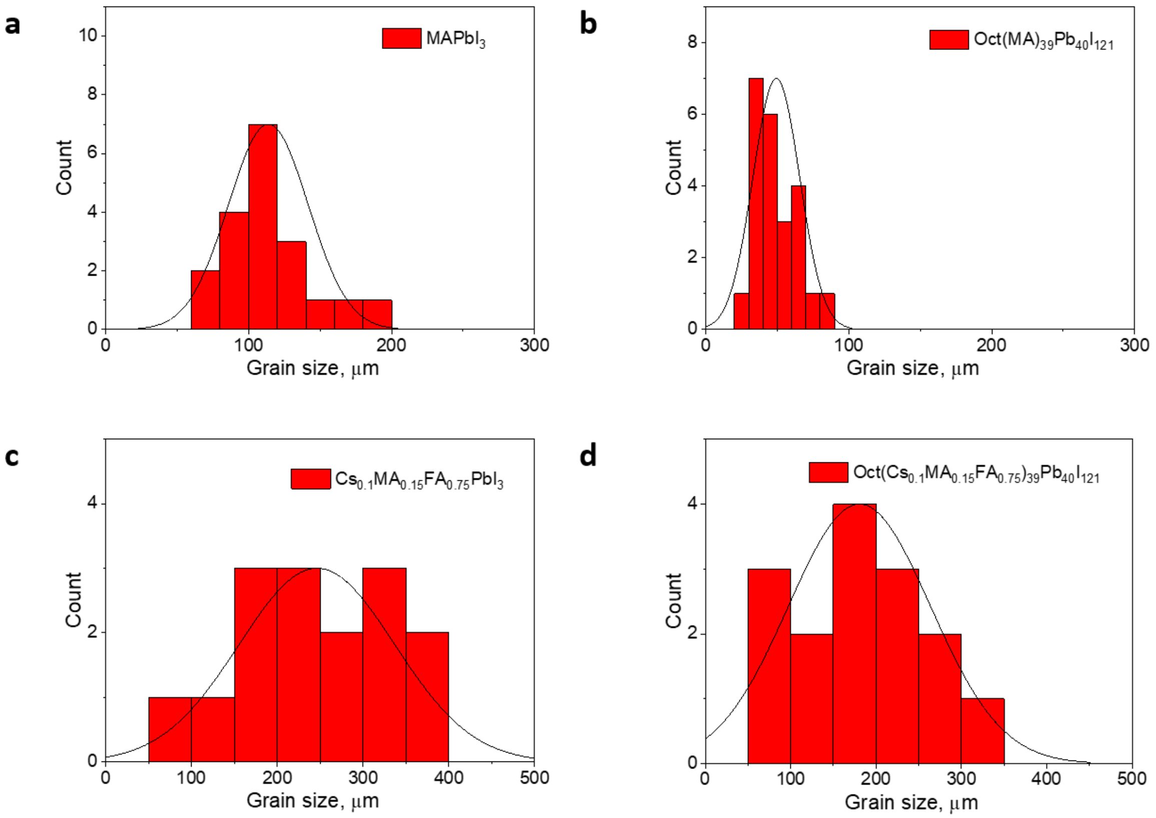

Figure S4. Grain size distribution for the pristine $MAPbI_3$ (a), $Cs_{0.1}MA_{0.15}FA_{0.75}PbI_3$ (c), modified $Oct(MA)_{39}Pb_{40}I_{121}$ (b), and $Oct(Cs_{0.1}MA_{0.15}FA_{0.75})_{39}Pb_{40}I_{121}$ (d) films.

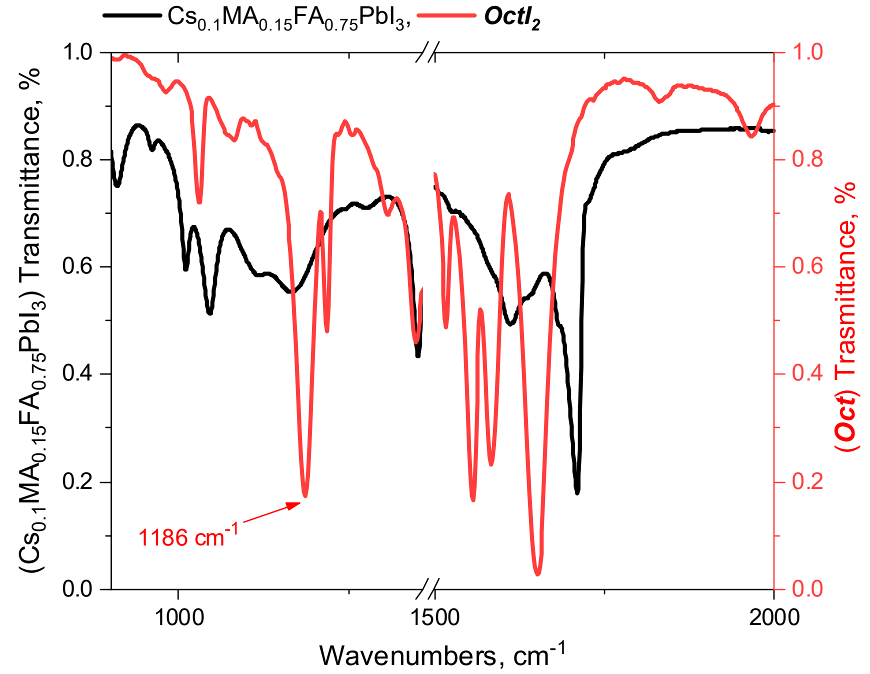

Figure S5. FTIR spectrum of the $Cs_{0.1}MA_{0.15}FA_{0.75}PbI_3$ and octenidine dihydroiodide powders.

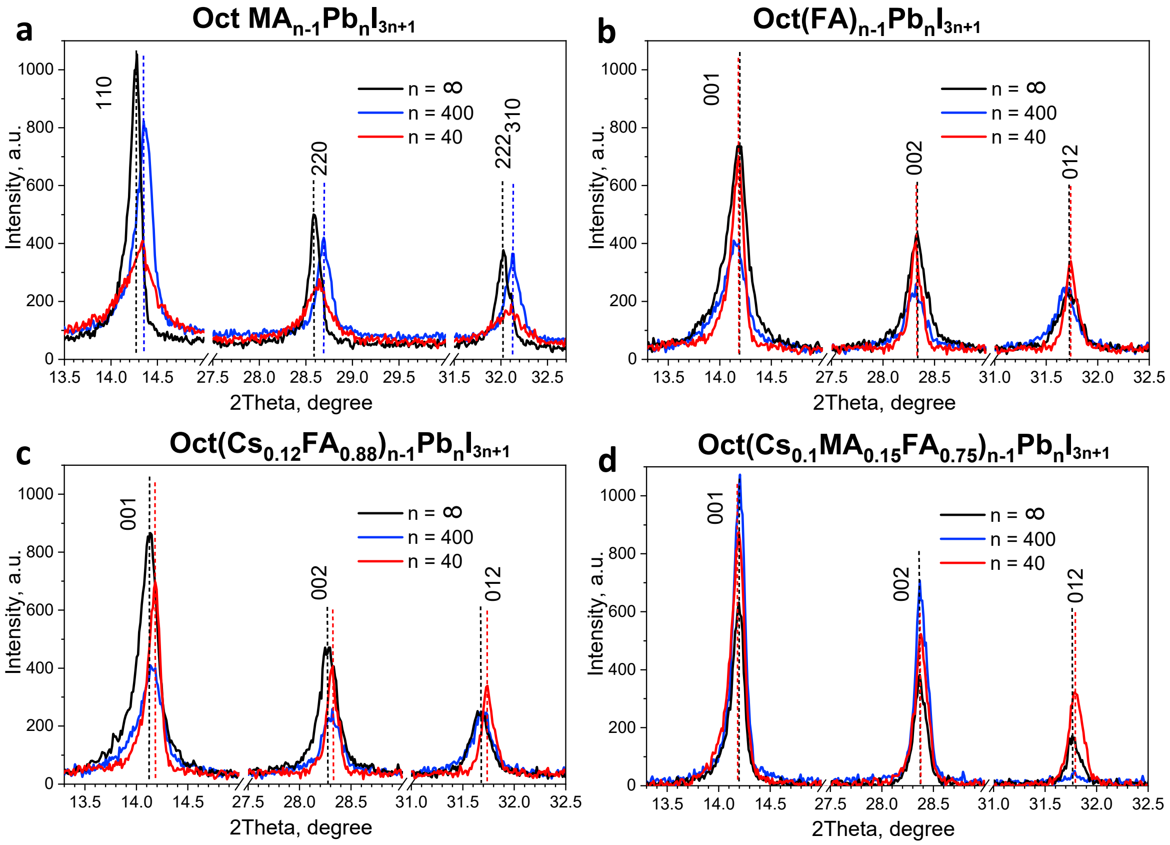

Figure S6. The zoomed parts of the diffraction patterns for the Oct(A)$_{n-1}$Pb$_n$I$_{3n+1}$ films with different ***OctI$_2$*** loadings compared to the reference APbI$_3$ samples, where A = MA (a), FA (b), Cs$_{0.12}$FA$_{0.88}$ (c), and Cs$_{0.1}$MA$_{0.15}$FA$_{0.75}$ (d).

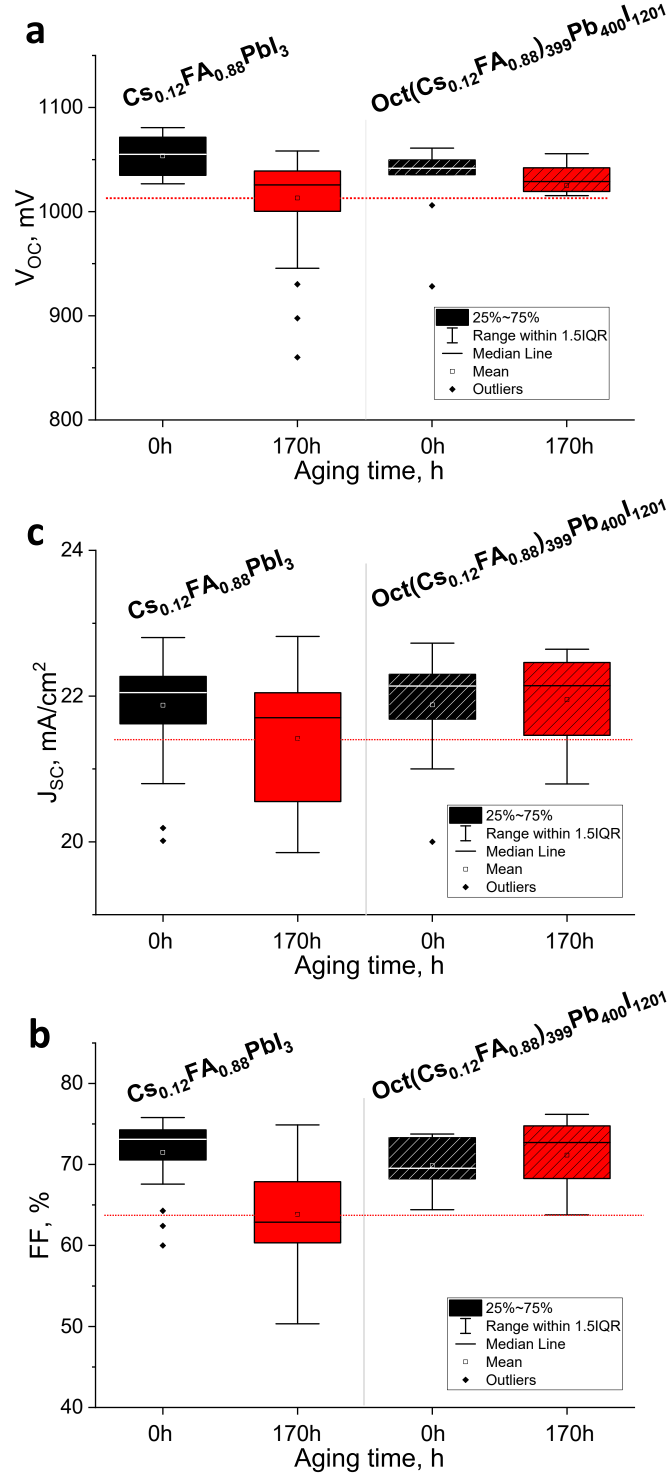

Figure S7. The open circuit voltage (V_{OC}) (a), short circuit current density (J_{SC}) (b), and fill factor values (FF) (c) of the ITO/PTA/Perovskite/PCBM/Al devices based on $Cs_{0.12}FA_{0.88}PbI_3$ and $Oct(Cs_{0.12}FA_{0.88})_{399}Pb_{400}I_{1201}$ absorber films with and w/o exposure to light for 170 h (70 mW/cm^2, T= 38±3°C).

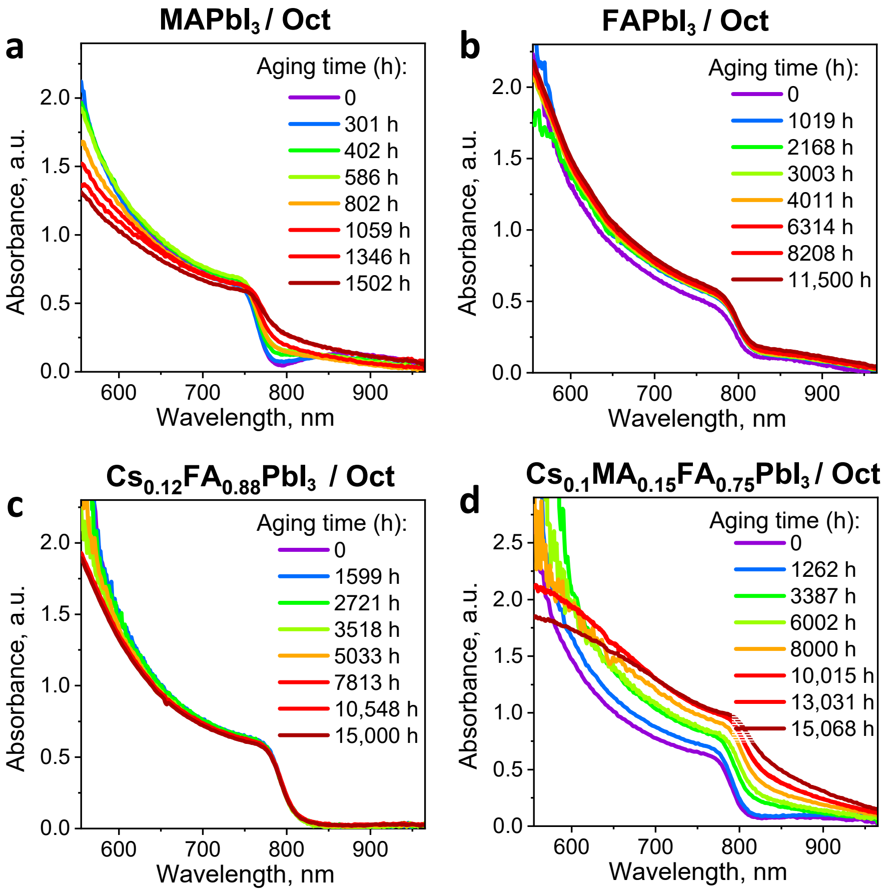

Figure S8. The evolution of the UV-Vis spectra of the $APbI_3$ films modified by ***OctI₂***-free surface coating upon light exposure for A = MA (a), FA (b), $Cs_{0.12}FA_{0.88}$ (c), and $Cs_{0.1}MA_{0.15}FA_{0.75}$ (d).

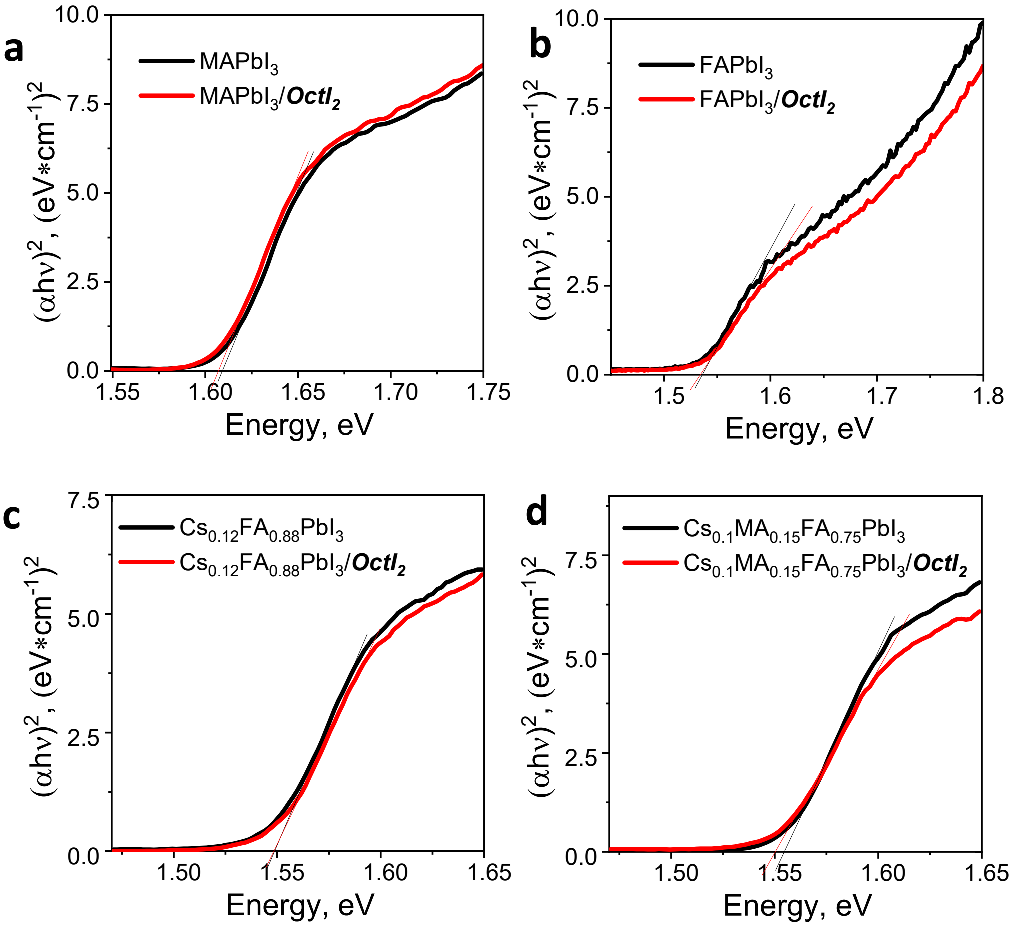

Figure S9. Tauc plots for $APbI_3$ films modified by the ***OctI***$_2$ surface coating used for E_g estimation (assuming direct bandgap) for A = MA (a), FA (b), $Cs_{0.12}FA_{0.88}$ (c), and $Cs_{0.1}MA_{0.15}FA_{0.75}$ (d).

Table S1. Overview of the relevant reported data on the perovskite solar cells based on the Dion-Jacobson absorber materials.

Bulk cation or corresponding amine	Light absorber	Device structure	PCE, %	Aging conditions	Aging time / Retained fraction (%) of the initial efficiency	Ref.
Ethylenediamine dihydrochloride ($EDACl_2$)	$EDACl_2$ / $Cs_{0.15}FA_{0.85}PbI_{2.7}Br_{0.3}$ 2D/3D	ITO/SnO_2/$Cs_{0.15}FA_{0.85}PbI_{2.7}Br_{0.3}$/Spiro-MeOTAD/Ag	**19.68**	**N/A**	**N/A**	[1]
Ethane-1,2-diammonium iodide ($EDAI_2$)	$MA_{1-2x}EDA_xPbI_3$ 2D/3D	FTO/c-TiO_2/Perovskite/spiro-OMeTAD/Au	**17.0**	Light (1 sun), T=50 °C, RH~50%,	72h / 75%	[2]
Ethane-1,2-diammonium iodide ($EDAI_2$)	$CsPbI_3$·xEDAPbI4	FTO/c-TiO2/Perovskite/spiro-OMeTAD/Ag	**11.86**	Dark, RT, inert atmosphere	840 h / 75%	[3]
Ethane-1,2-diammonium iodide ($EDAI_2$)	$EDAI_2$/$FASnI_3$ 2D/3D	ITO/PEDOT:PSS/PSK/C_{60}/BCP/Ag	**8.9**	Dark, RT, ambient air (encapsulated)	2000 h / 100%	[4]
N,N'-dimethylethylene-1,2-diammonium iodide	$DEAI_2$/$MAPbI_3$ 2D/3D		**20.2**	Dark, RT, RH ~60-80 % (unencapsulated)	720 h / 73.7%	[5]
1,3-propanediammmonium iodide (PRI2)	$(PR)_2(FA)_8Pb_9I_{28}$	FTO/c-TiO2/Perovskite/spiro-OMeTAD/Au	**12.92**	Dark, RT, RH ~80 %	500 h / 50%	[6]

1,4-butanediammonium iodide (BUI2)	$(BU)_2(FA)_8Pb_9I_{28}$	FTO/c-TiO_2/Perovskite/spiro-OMeTAD/Au	**11.71**	Dark, RT, RH ~80 %	500 h / 30%	
1,6-Diaminohexane Dihydrochloride (1,6-DD)	1,6-DD /$MAPbI_3$<br>2D/3D		**17.0**	Dark, ambient air, RH~10-30% (unencapsulated)	384 h / 30%	[7]
1,8-octanediammonium iodide (ODAI)	1,8-OD /$FAPbI_3$<br>2D/3D	(ITO/SnO_2/perovskite/PCBM/Ag	**21.18**	Dark, ambient air (RH~20-40%, RT) (unencapsulated)	884 h / 92%	[8]
1,8-octanediammonium iodide (ODAI)	$NH_3I(CH_2)_8NH_3I$ /$MAPbI_3$<br>2D/3D	ITO/Cu:NiO_x/perovskite/PCBM/bis-C_{60}/Ag	**17.6**	N/A	N/A	[9]
2,2-(ethylenedioxy)bis(ethylammonium iodide) (EDBEI)	$(EDBEPbI_4)_{0.03}[Cs_{0.05}(FA_{0.83}MA_{0.17})_{0.95}Pb(I_{0.83}Br_{0.17})_3]_{0.97}$	ITO/c-TiO_2/perovskite/Spiro-OMeTAD/Au	**21.6**	Dark, ambient air, RH~65%)<br>---<br>Light (1 sun), MPPT, N_2 atmosphere (unencapsulated)<br>---<br>Dark, ambient air, RH~40-50%) (encapsulated)	1000 h / 65%<br>---<br>500 h / 95%<br>---<br>3000 h / 95%	[10]
p-phenylenediammonium (PPE)	PPE /$Cs_{0.06}FA_{0.79}MA_{0.15}PbI_{2.55}Br_{0.45}$<br>2D/3D	ITO/c-TiO_2/perovskite/Spiro-OMeTAD/Au	**16.03**	NA	NA	[11]
Bidentate ligand 2,2-bipyridine (Bpy)	Bpy /$FA_{0.88}Cs_{0.12}PbI_3$<br>2D/3D	ITO/c-TiO2/perovskite/Spiro-OMeTAD/Au	**18.6**	Light (1 sun), N_2 atmosphere, RT (unencapsulated)<br>---<br>Dark, inert atmosphere, T = 85 °C) (unencapsulated)<br>---<br>Dark, ambient air, RH~50%, RT (unencapsulated	240 h / 100%<br>--<br>196 h / 77%<br>--<br>670 h / 43%	[12]
Pyrazine			**20.58**	Dark, ambient air, RH~55%, RT	50 h / 90%	[13]
imidazolium iodide	imidazolium iodide / $MAPbI_3$<br>2D/3D		**19.9**	Light, MPPT (unencapsulated)	300 h / 90%	[14]
2-Methylimidazole	$(FAPbI_3)_{1-x}(MAPbBr_3)_x$<br>2D/3D		**19.45**	N/A	N/A	[15]
(aminomethyl)pyridinium (3AMPY)	$(3AMP)(MA)_3Pb_4I_{13}$	FTO/PEDOT:PSS/perovksite/C_{60}/BCP/Ag	7.32	N/A	N/A	[16]

(aminomethyl)pyridinium (3AMPY)	$(3AMP)(MA_{0.75}FA_{0.25})_3Pb_4I_{13}$	FTO/PEDOT:PSS/perovskite/C_{60}/BCP/Ag	12.04	Light in ambient air (50–70% RH) (unencapsulated)	48 h / 22 %	[17]
3-(aminomethyl)pyridinium (3AMPY)	$(3AMP)(MA_{0.75}FA_{0.25})_3Pb_4I_{13}$	ITO/PTAA/PFN/perovskite/$PC_{61}CM$/BCP/Ag	16.25	Dark, ambient atmosphere (25 ± 5% RH)<br>(45 ± 5% RH) (unencapsulated)	2304 h / 90%<br>840 h / 80%	[18]
3-(aminomethyl)pyridinium (3AMPY)	$3AMPY(MA)_3Pb_4I_{13}$	FTO/PEDOT:PSS/perovksite/C_{60}/BCP/Ag	9.20	N/A	N/A	[19]
4-aminomethyl)pyridinium (4AMPY)	$(4AMP)(MA)_2Pb_3I_{10}$	FTO/PEDOT:PSS/perovskite/C_{60}/BCP/Ag	3.67	N/A	N/A	[16]
4-aminomethyl)pyridinium (4AMP)	$(4AMP)(FA)_3Sn_4I_{13}$	FTO/TiO_2/ZrO_2/perovskite/C	4.22	1-sun illumination N_2 atmosphere, T=45 °C (unencapsulated)	100 h / 91%	[17]
3- (aminomethyl)piperidinium (3AMP)	$3AMP/(MA_{0.5}FA_{0.5})_3(Pb_{0.5}Sn_{0.5})_4I_{13}$	ITO/PEDOT:PSS/perovskite/PCBM/BCP/Ag	20.09	Continuous illumination in ambient air (20–50% RH, RT) (encapsulated)	100 h / 73.2%	[18]
4-(aminomethyl)pyridinium (4AMPY)	$(4AMPY)(MA)_3Pb_4I_{13}$	FTO/PEDOT:PSS/perovksite/C_{60}/BCP/Ag	5.69	N/A	N/A	[19]
2,5-thiophenedimethylammonium (ThDMA)	$(ThDMA)MA_4Pb_5I_{16}$	ITO/PEDOT: PSS/perovskite/PCBM/BCP/Ag	15.75	Dark, N_2 atmosphere, RT<br>Continuous illumination in N_2 atmosphere,<br>Dark, T=80 °C, N_2 atmosphere. (unencapsulated)	1655 h / >95%<br>668 h / 88%<br>…h / 90%	[20]
3-(dimethylammonium)-1-propylammonium (DMAPA)	$(DMAPA)PbI_4$	FTO/TiO_2/perovskite/spiro-OMeTAD/Au	3.85	N/A	N/A	[21]
3-(dimethylammonium)-1-propylammonium (DMAPA)	$(DMAPA)MA_3Pb_4I_{13}$	FTO/TiO_2/perovskite/spiro-OMeTAD/Au	15.16	Dark, T=85 °C in air;<br>Continuous illumination (unencapsulated)	1000 h / >90%<br>300 h / 80%	[21]
3-(dimethylammonium)-1-propylammonium (DMAPA)	$(DMAPA)MA_4Pb_5I_{16}$	FTO/TiO_2/perovskite/spiro-OMeTAD/Au	12.41	N/A	N/A	[21]
1,3-propanediammonium	$(PDA)MAPb_2I_7$	FTO/TiO_2/perovskite/Spiro-OMeTAD/Au	1.27	N/A	N/A	[22]
1,3-propanediammonium	$(PDA)MA_2Pb_3I_{10}$	FTO/TiO_2/perovskite/Spiro-OMeTAD/Au	9.10	N/A		[22]

1,3-propanediammonium	$(PDA)MA_3Pb_4I_{13}$	FTO/TiO_2/perovskite/Spiro-OMeTAD/Au	13.3	>95% under various harsh environmental stresses,<br><br>including in ambient air with 40–70% RH after 4000 h,<br><br>in damp heat condition with 85 °C and 85% RH after 168 h, and in continuous illumination for 3000 h. (unencapsulated)		[22]
1,3-propanediammonium	$(PDA)MA_2Pb_3I_{10}$	ITO/PEDOT:PSS/perovksite/C_{60}/BCP/Ag	9.0	N/A	N/A	[23]
1,3-propanediammonium	$(PDA)FA_2Pb_3I_{10}$	ITO/SnO_2/perovskite/Spiro-OMeTAD/Ag	8.45	Dark, T= 85 °C in the N_2 atmosphere (unencapsulated)	100 h/ 50%	[23]
1,3-propanediammonium	$(PDA)FA_3Pb_4I_{13}$	ITO/SnO_2/perovskite/Spiro-OMeTAD/Ag	13.8	Dark, T= 85 °C in the N_2 atmosphere (unencapsulated)<br><br>Dark, under ambient condition (30–40% RH, RT) (unencapsulated)	1000 h / 85%<br><br>540 h / 85%	[23]
propane-1,3-diammonium	$(PDA)MA_2Pb_3I_{10}$	ITO/PEDOT:PSS/perovskite/$PC_{61}BM$/BCP/Cu	11.3	N/A	N/A	[23]
1.3-propanediamine	$PDAMA_4Pb_5I_{16}$	ITO/PEDOT:PSS/perovskite/$PC_{60}BM$/LiF/Al	14.16	Dark, under ambient condition (45% RH, RT) (unencapsulated)	480 h / 30%	[24]
1,4-butanediammonium	$BDAPbI_4$	FTO/TiO_2/perovskite/Spiro-OMeTAD/Ag	1.08	Dark, under ambient condition	96 h / 73.9%	[25]
1,4-butanediammonium	$BDAPbI_4$	FTO/c-TiO_2/mp-TiO_2/perovskite<br><br>/Spiro-OMeTAD/Au	1.1	N/A	N/A<br>.	[26]
1,4-butanediammonium	$BDAMA_4Pb_5I_{16}$	ITO/PTAA/perovskite/PCBM/BCP/Ag	14.53	Dark, (50 ± 5% RH, RT) (encapsulated)	900 h / 85%	[27]
1,4-butanediammonium	$BDAMA_4Pb_5I_{16}$	ITO/PEDOT:PSS/perovskite/$PC_{60}BM$/LiF/Al	16.38	Dark, ambient air (45% RH, RT) (unencapsulated)	480 h / 80%	[24]
1,4-butanediammonium	$BDAMA_3Pb_5I_{16}$	ITO/PEDOT:PSS/perovskite/$PC_{60}BM$/LiF/Au	17.91	Dark, ambient air (60% RH, RT)<br><br>Light, (unencapsulated)	1182h / 84%<br><br>298 h / 90%	[28]
1,4-butanediammonium	$(BDA)(Cs_{0.1}FA_{0.9})_4Pb_5I_{16}$	FTO/TiO_2/perovskite/Spiro-OMeTAD/Ag	18.2	Dark, 80% RH (unencapsulated)<br><br>Dark, T= 85 °C (unencapsulated)	800 h / 100%<br><br>800 h / 80%<br><br>800 h / 90%<br><br>.	[29]

				Continuous illumination at MPP (encapsulated)		
1,4-butanediammonium	(BDA)$FA_2Sn_3I_{10}$	ITO/PEDOT:PSS/perovskite/PCBM/Ag	6.43	Dark, N_2 environment, RT<br>Dark, (40% RH, RT)<br>Dark, T = 100 °C (unencapsulated)	1000 h / 92%<br>200 h / 89%<br>50 h / 82%	[30]
1,4-butanediammonium	(BDA)$MA_3Pb_4I_{13}$	(PEDOT:PSS/ perovskite/ bathocuproine/Ag)	12.81	N/A	N/A	[31]
1,4-butanediammonium	(BDA)(MA0.07FA0.9 3)4Pb5I16	ITO/SnO2/Perovskite /Spiro-OMeTAD/Au	19.55	N/A	N/A	[32]
1,4-butanediammonium	(BDA)$FA_4Pb_5I_{16-x}Br_x$	ITO/SnO_2/perovskite/Spiro-OMeTAD/Au	16.75	Dark, 15–20% RH,<br>Dark, T = 60 °C	1600 h / 93%<br>400 h / 91%	[33]
(4F-PhDMA)	(4F-PhDMA) (MA)3Pb4I13	ITO/PEDOT:PSS/Perovskite/PCBM/BCP/Ag	17.37	N/A	N/A	[34]
Pentamethylenediamine (PeDA)	$PeDAMA_4Pb_5I_{16}$	ITO/PEDOT:PSS/perovskite/$PC_{60}BM$/LiF/Al	12.95	Dark, ambient air (45% RH) (unencapsulated)	480 h / ~100%	[24]
1,6-diaminohexane dihydroiodide	$HDAPbI_4$	FTO/TiO_2/perovskite/Spiro-OMeTAD/Ag	0.592	Dark, ambient condition	96 h / 57.4%	[25]
1,6-diaminohexane dihydroiodide	$HDAMA_3Pb_4I_{13}$	ITO/SnO_2/perovskite/Spiro-OMeTAD/Au	7.35	Dark, 50–60% RH<br>Continuous illumination at MPP<br>Dark, T = 60 °C<br>Dark, T = 70 °C (encapsulated).	1248 h / 41.3%<br>4.5 h / 69.6%<br>68h / 60.2%<br>68 h / 54.9%	[35]
1,6-diaminohexane dihydroiodide	$HDAMA_4Pb_5I_{16}$	ITO/PEDOT:PSS/perovskite/$PC_{60}BM$/LiF/Al	10.55	Dark, ambient air (45% RH)	480 h / 60%	[24]
1,8-diaminooctane dihydroiodide	$ODAPbI_4$	FTO/TiO2/perovskite/Spiro-OMeTAD/Ag	0.01	N/A	N/A	[25]
TTDMA	(TTDMA)(MA)3Pb4 I13	ITO/PEDOT:PSS/Perovskite/PCBM/BCP/Ag	18.82	Dark, N_2	4400 h / 99%	[36]
trans-1,4-cyclohexanediamine dihydroiodide	(CHDA)PbI_4	FTO/SnO_2/perovskite/Spiro-OMeTAD/Au	0.52	N/A	N/A	[35]
trans-1,4-cyclohexanediamine dihydroiodide	(CHDA)$MAPb_2I_7$	FTO/SnO_2/perovskite/Spiro-OMeTAD/Au	2.89	N/A	N/A	[35]
trans-1,4-cyclohexanediamine dihydroiodide	(CHDA)$MA_2Pb_3I_{10}$	FTO/SnO_2/perovskite/Spiro-OMeTAD/Au	6.2	N/A	N/A	[35]
trans-1,4-cyclohexanediamine dihydroiodide	(CHDA)$MA_3Pb_4I_{13}$	ITO/SnO_2/perovskite/Spiro-OMeTAD/Au	15.01	Dark, (50–60% RH)<br>Continuous illumination at MPP	1248 h / 94.7%<br>4.5h / 80.7%	[35]

				Dark, T= 60 °C<br><br>Dark, T= 60 °C (encapsulated).	68h / 96.5%<br><br>68h / 74.4%	
p-phenylenediamine dihydroiodide	(PPD)$(MA)_2Pb_3I_{10p-}$	ITO/PEDOT:PSS/perovskite/$PC_{61}BM$/BCP/Cu	12.3	N/A	N/A	[37]
1,4- phenylenedimethylammonium	(PDMA)PbI_4	FTO/c-TiO_2/mp-TiO_2/perovskite<br><br>/Spiro-OMeTAD/Au	0.91	N/A	N/A	[38]
1,4- phenylenedimethylammonium	(PDMA)$FAPb_2I_7$	FTO/c-TiO_2/mp-TiO_2/perovskite<br><br>/Spiro-OMeTAD/Au	2.91	N/A	N/A	[38]
1,4- phenylenedimethylammonium	(PDMA)$FA_2Pb_3I_{10}$	FTO/c-TiO_2/mp-TiO_2/perovskite<br><br>/Spiro-OMeTAD/Au	7.11	Dark, ambient air (30–50% RH, RT)<br><br>MPP tracking under continuous illumination, T = 60 °C in N_2 atmosphere (unencapsulated)	1440 h / 85%<br><br>500 h / 60%	[38]
1,4- phenylenedimethylammonium	(PDMA)$(MA)_2Pb_3I_{10}$	ITO/PEDOT:PSS/perovskite/$PC_{61}BM$/BCP/Cu	15.6	MPP tracking under continuous illumination, N_2 atmosphere<br><br>Dark, N_2 atmosphere. (unencapsulated)	700 h / 90%<br><br>1500 h / 90%	[37]
1,4- phenylenedimethylammonium	(PDMA)$FA_3Pb_4I_{13}$	FTO/c-TiO_2/mp-TiO_2/perovskite<br><br>/Spiro-OMeTAD/Au	6.52	N/A	N/A	[38]
1,4- phenylenedimethylammonium	(PDMA)$(Cs_{0.05}MA_{0.15}FA_{0.8})_9Pb_{10}(I_{0.93}Br_{0.07})_{31}$	FTO/c-TiO_2/mp-TiO_2/perovskite<br><br>/Spiro-OMeTAD/Au	15.6	Dark, ambient air (20–50% RH) . (unencapsulated)	84 h / 220%	[37]
1,4-benzenedimethylammonium iodide (BzDAI)	(BzDA)-(Cs0.05MA0.15FA0.8)9Pb10(I0.93Br0.07)31	FTO/c-TiO_2/mp-TiO_2/perovskite<br><br>/Spiro-OMeTAD/Au	15.6	Dark, ambient air (50% RH) (unencapsulated)	160 h / 60%	[39]
N-(3-aminopropyl)-2-pyrrolidinone (NAP)	NAP/$MASn_{0.5}Pb_{0.5}I_xCl_3$ $_{2D/3D}$	ITO/PEDOT:PSS/perovskite/$PC_{61}BM$/Bhen-NaDPO/Ag	13.4	Dark, ambient conditions (RH 30 ± 5%) (unencapsulated)	720 h / 90%	[40]

Table S2. Experimental E*g* values and the absorption edge (in brackets) for the $OctA_{n-1}Pb_nI_{3n+1}$ films of various compositions obtained from the corresponding Tauc plots.

**Perovskite composition**	**Eg, eV**		
	**n = ∞ (pristine)**	**n = 400**	**n = 40**
$OctMA_{n-1}Pb_nI_{3n+1}$	1.608 (771 nm)	1.612 (769 nm)	1.633 (759 nm)
$OctFA_{n-1}Pb_nI_{3n+1}$	1.535 (808 nm)	1.529 (811 nm)	1.525 (813 nm)
$Oct(Cs_{0.12}FA_{0.88})_{n-1}Pb_nI_{3n+1}$	1.546 (802 nm)	1.550 (800 nm)	1.555 (797 nm)
$Oct(Cs_{0.1}MA_{0.15}FA_{0.75})_{n-1}Pb_nI_{3n+1}$	1.555 (797 nm)	1.552 (799 nm)	1.545 (803 nm)

Table S3. Average and the best (in brackets) values of the device parameters based on $Oct(Cs_{0.12}FA_{0.88})_{n-1}Pb_nI_{3n+1}$ absorber layers with different ***OctI***$_2$ contents.*.

		$Oct(Cs_{0.12}FA_{0.88})_{n-1}Pb_nI_{3n+1}$			
**n =**	**Scan direction**	V_{OC}**, mV**	J_{SC}**, mA/cm²**	**FF, %**	**PCE, %**
∞ (pristine)	Forward	1041±15 (1056)	21.4±0.3 (21.7)	72.4±3.2 (75.6)	16.3±1.0 (17.3)
	Reverse	1045±16 (1061)	21.3±0.4 (21.7)	73.1±3.9 (77.0)	16.5±1.2 (17.7)
400	Forward	1019±12 (1031)	20.9±0.4 (21.3)	71.9±3.3 (75.2)	15.3±1.2 (16.5)
	Reverse	1024±11 (1035)	21.0±0.3 (21.3)	75.4±3.7 (79.1)	16.2±1.2 (17.4)
200	Forward	841±17 (858)	17.5±0.7 (18.2)	50.8±5.0 (55.8)	7.5±1.2 (8.7)
	Reverse	862±18 (880)	17.0±0.6 (17.6)	56.2±4.8 (61.0)	8.3±1.1 (9.4)
40	Forward	679±21 (700)	10.2±0.9 (11.1)	46.8±5.0 (51.8)	3.4±0.7 (4.1)
	Reverse	704±19 (723)	9.7±1.1 (10.8)	55.2±4.9 (60.1)	3.8±0.9 (4.7)

Notes: * the *J–V* curves were measured in forward directions with a scan rate of 0.01 V/s.

References

1. 43. Hou, J.; Deng, F.; Wu, Q.; Yang, L.; Wu, J.; Li, X.; Zheng, Y.-Z.; Li, N.; Ding, H.; Tao, X. Ethylenediamine Chlorides Additive Assisting Formation of High-Quality Formamidinium-Caesium Perovskite Film with Low Trap Density for Efficient Solar Cells. Journal of Power Sources 2020, 449, 227484. https://doi.org/10.1016/j.jpowsour.2019.227484.
2. 21. Lu, J.; Jiang, L.; Li, W.; Li, F.; Pai, N. K.; Scully, A. D.; Tsai, C.; Bach, U.; Simonov, A. N.; Cheng, Y.; Spiccia, L. Diammonium and Monoammonium Mixed-Organic-Cation Perovskites for High Performance Solar Cells with Improved Stability. Advanced Energy Materials 2017, 7(18). https://doi.org/10.1002/aenm.201700444.
3. 44. Zhang, T.; Dar, M. I.; Li, G.; Xu, F.; Guo, N.; Grätzel, M.; Zhao, Y. Bication Lead Iodide 2D Perovskite Component to Stabilize Inorganic A-CsPbI 3 Perovskite Phase for High-Efficiency Solar Cells. Science Advances 2017, 3 (9). https://doi.org/10.1126/sciadv.1700841.
4. 45. Jokar, E.; Chien, C.-H.; Fathi, A.; Rameez, M.; Chang, Y.-H.; Diau, E. W.-G. Slow Surface Passivation and Crystal Relaxation with Additives to Improve Device Performance and Durability for Tin-Based Perovskite Solar Cells. Energy & Environmental Science 2018, 11 (9), 2353–2362. https://doi.org/10.1039/C8EE00956B.
5. 22. He, Q.; Worku, M.; Xu, L.; Zhou, C.; Lin, H.; Robb, A. J.; Hanson, K.; Xin, Y.; Ma, B. Facile Formation of 2D–3D Heterojunctions on Perovskite Thin Film Surfaces for Efficient Solar Cells. ACS Applied Materials & Interfaces 2020, 12 (1), 1159–1168. https://doi.org/10.1021/acsami.9b17851.
6. 46. Zheng, H.; Liu, G.; Zhu, L.; Ye, J.; Zhang, X.; Alsaedi, A.; Hayat, T.; Pan, X.; Dai, S. The Effect of Hydrophobicity of Ammonium Salts on Stability of Quasi-2D Perovskite Materials in Moist Condition. Advanced Energy Materials 2018, 8 (21). https://doi.org/10.1002/aenm.201800051.
7. 47. Wang, Y.; Liu, S.; Zeng, Q.; Wang, R.; Qin, W.; Cao, H.; Yang, L.; Li, L.; Yin, S.; Zhang, F. Enhanced Performance and Stability of Inverted Planar Perovskite Solar Cells by Incorporating 1,6-Diaminohexane Dihydrochloride Additive. Solar Energy Materials and Solar Cells 2018, 188, 140–148. https://doi.org/10.1016/j.solmat.2018.07.031.
8. 48. Luo, W.; Wu, C.; Wang, D.; Zhang, Y.; Zhang, Z.; Qi, X.; Zhu, N.; Guo, X.; Qu, B.; Xiao, L.; Chen, Z. Efficient and Stable Perovskite Solar Cell with High Open-Circuit Voltage by Dimensional Interface Modification. ACS Applied Materials & Interfaces 2019, 11 (9), 9149–9155. https://doi.org/10.1021/acsami.8b22040.
9. 49. Zhao, T.; Chueh, C.-C.; Chen, Q.; Rajagopal, A.; Jen, A. K.-Y. Defect Passivation of Organic–Inorganic Hybrid Perovskites by Diammonium Iodide toward High-Performance Photovoltaic Devices. ACS Energy Letters 2016, 1 (4), 757–763.https://doi.org/10.1021/acsenergylett.6b00327.
10. 50. Li, P.; Zhang, Y.; Liang, C.; Xing, G.; Liu, X.; Li, F.; Liu, X.; Hu, X.; Shao, G.; Song, Y. Phase Pure 2D Perovskite for High-Performance 2D–3D Heterostructured Perovskite Solar Cells. Advanced Materials 2018, 30 (52). https://doi.org/10.1002/adma.201805323.
11. 51. Xin, D.; Tie, S.; Yuan, R.; Zheng, X.; Zhu, J.; Zhang, W.-H. Defect Passivation in Hybrid Perovskite Solar Cells by Tailoring the Electron Density Distribution in Passivation Molecules. ACS Applied Materials & Interfaces 2019, 11 (47), 44233–44240.https://doi.org/10.1021/acsami.9b15166.
12. 52. Chen, J.; Kim, S.-G.; Ren, X.; Jung, H. S.; Park, N.-G. Effect of Bidentate and Tridentate Additives on the Photovoltaic Performance and Stability of Perovskite Solar Cells. Journal of Materials Chemistry A 2019, 7 (9), 4977–4987. https://doi.org/10.1039/C8TA11977E.
13. 53. Lee, M.-S.; Sarwar, S.; Park, S.; Asmat, U.; Thuy, D. T.; Han, C.; Ahn, S.; Jeong, I.; Hong, S. Efficient Defect Passivation of Perovskite Solar Cells via Stitching of an Organic Bidentate Molecule. Sustainable Energy & Fuels 2020, 4 (7), 3318–3325.https://doi.org/10.1039/C9SE01041F.\
14. 54. Salado, M.; Jodlowski, A. D.; Roldan-Carmona, C.; de Miguel, G.; Kazim, S.; Nazeeruddin, M. K.; Ahmad, S. Surface Passivation of Perovskite Layers Using Heterocyclic Halides: Improved Photovoltaic Properties and Intrinsic Stability. Nano Energy 2018, 50, 220– 228. https://doi.org/10.1016/j.nanoen.2018.05.035.

15. 55. Shen, D.; Wu, W.; Li, Y.; Abate, A.; Wei, M. 2-Methylimidazole as an Interlayer for the Enhancement of the Open-Circuit Voltage in Perovskite Solar Cells. Journal of Power Sources 2020, 450, 227714. https://doi.org/10.1016/j.jpowsour.2020.227714.
16. 30. Mao, L.; Ke, W.; Pedesseau, L.; Wu, Y.; Katan, C.; Even, J.; Wasielewski, M. R.; Stoumpos, C. C.; Kanatzidis, M. G. Hybrid Dion–Jacobson 2D Lead Iodide Perovskites. Journal of the American Chemical Society 2018, 140 (10), 3775–3783. https://doi.org/10.1021/jacs.8b00542.
17. 56. Ke, W.; Mao, L.; Stoumpos, C. C.; Hoffman, J.; Spanopoulos, I.; Mohite, A. D.; Kanatzidis, M. G. Compositional and Solvent Engineering in Dion–Jacobson 2D Perovskites Boosts Solar Cell Efficiency and Stability. Advanced Energy Materials 2019, 9 (10). https://doi.org/10.1002/aenm.201803384.
18. 57. Wu, H.; Lian, X.; Tian, S.; Zhang, Y.; Qin, M.; Zhang, Y.; Wang, F.; Lu, X.; Wu, G.; Chen, H. Additive-Assisted Hot-Casting Free Fabrication of Dion–Jacobson 2D Perovskite Solar Cell with Efficiency Beyond 16%. Solar RRL 2020, 4 (7).https://doi.org/10.1002/solr.202000087.
19. 58. Li, X.; Ke, W.; Traoré, B.; Guo, P.; Hadar, I.; Kepenekian, M.; Even, J.; Katan, C.; Stoumpos, C. C.; Schaller, R. D.; Kanatzidis, M. G. Two-Dimensional Dion–Jacobson Hybrid Lead Iodide Perovskites with Aromatic Diammonium Cations. Journal of the American Chemical Society 2019, 141 (32), 12880–12890. https://doi.org/10.1021/jacs.9b06398.
20. 59. Lu, D.; Lv, G.; Xu, Z.; Dong, Y.; Ji, X.; Liu, Y. Thiophene-Based Two-Dimensional Dion–Jacobson Perovskite Solar Cells with over 15% Efficiency. Journal of the American Chemical Society 2020, 142 (25), 11114–11122. https://doi.org/10.1021/jacs.0c03363.
21. 60. Zhao, W.; Dong, Q.; Zhang, J.; Wang, S.; Chen, M.; Zhao, C.; Hu, M.; Jin, S.; Padture, N. P.; Shi, Y. Asymmetric Alkyl Diamine Based Dion–Jacobson Low-Dimensional Perovskite Solar Cells with Efficiency Exceeding 15%. Journal of Materials Chemistry A 2020, 8 (19),9919–9926. https://doi.org/10.1039/D0TA02706E.
22. 31. Ahmad, S.; Fu, P.; Yu, S.; Yang, Q.; Liu, X.; Wang, X.; Wang, X.; Guo, X.; Li, C. Dion-Jacobson Phase 2D Layered Perovskites for Solar Cells with Ultrahigh Stability. Joule 2019, 3(3), 794–806. https://doi.org/10.1016/j.joule.2018.11.026.
23. 32. Ma, C.; Shen, D.; Ng, T.; Lo, M.; Lee, C. 2D Perovskites with Short Interlayer Distance for High-Performance Solar Cell Application. Advanced Materials 2018, 30 (22). https://doi.org/10.1002/adma.201800710.
24. 29. Li, F.; Zhang, J.; Jo, S.; Qin, M.; Li, Z.; Liu, T.; Lu, X.; Zhu, Z.; Jen, A. K. -Y. Vertical Orientated Dion–Jacobson Quasi-2D Perovskite Film with Improved Photovoltaic Performance and Stability. Small Methods 2020, 4 (5). https://doi.org/10.1002/smtd.201900831.
25. 61. Zheng, Y.; Niu, T.; Qiu, J.; Chao, L.; Li, B.; Yang, Y.; Li, Q.; Lin, C.; Gao, X.; Zhang, C.; Xia, Y.; Chen, Y.; Huang, W. Oriented and Uniform Distribution of Dion–Jacobson Phase Perovskites Controlled by Quantum Well Barrier Thickness. Solar RRL 2019, 3 (9). https://doi.org/10.1002/solr.201900090.
26. 42. Safdari, M.; Svensson, P. H.; Hoang, M. T.; Oh, I.; Kloo, L.; Gardner, J. M. Layered 2D Alkyldiammonium Lead Iodide Perovskites: Synthesis, Characterization, and Use in Solar Cells. Journal of Materials Chemistry A 2016, 4 (40), 15638–15646. https://doi.org/10.1039/C6TA05055G.
27. 62. Safdari, M.; Phuyal, D.; Philippe, B.; Svensson, P. H.; Butorin, S. M.; Kvashnina, K. O.; Rensmo, H.; Kloo, L.; Gardner, J. M. Impact of Synthetic Routes on the Structural and Physical Properties of Butyl-1,4-Diammonium Lead Iodide Semiconductors. Journal of Materials Chemistry A 2017, 5 (23), 11730–11738. https://doi.org/10.1039/C6TA10123B.
28. 24. Niu, T.; Ren, H.; Wu, B.; Xia, Y.; Xie, X.; Yang, Y.; Gao, X.; Chen, Y.; Huang, W. Reduced-Dimensional Perovskite Enabled by Organic Diamine for Efficient Photovoltaics. The Journal of Physical Chemistry Letters 2019, 10 (10), 2349–2356. https://doi.org/10.1021/acs.jpclett.9b00750.
29. 63. Zhao, X.; Liu, T.; Kaplan, A. B.; Yao, C.; Loo, Y.-L. Accessing Highly Oriented Two-Dimensional Perovskite Films via Solvent-Vapor Annealing for Efficient and Stable Solar Cells. Nano Letters 2020, 20 (12), 8880–8889. https://doi.org/10.1021/acs.nanolett.0c03914.
30. 64. Li, P.; Liu, X.; Zhang, Y.; Liang, C.; Chen, G.; Li, F.; Su, M.; Xing, G.; Tao, X.; Song, Y. Low-Dimensional Dion–Jacobson-Phase Lead-Free Perovskites for High-Performance Photovoltaics with Improved Stability. Angewandte Chemie International Edition 2020, 59(17), 6909–6914. https://doi.org/10.1002/anie.202000460.
31. 65. Wang, H.; Chan, C. C. S.; Chu, M.; Xie, J.; Zhao, S.; Guo, X.; Miao, Q.; Wong, K. S.; Yan, K.; Xu, J. Interlayer Cross-Linked 2D Perovskite Solar Cell with Uniform Phase Distribution and Increased Exciton Coupling. Solar RRL 2020, 4 (4). https://doi.org/10.1002/solr.201900578.
32. 66. Jin, L.; Ren, N.; Wang, P.; Li, R.; Xue, Q.; Huang, F.; Zhang, X.; Zhao, Y.; Zhang, X. Secondary Anti-Solvent Treatment for Efficient 2D Dion–Jacobson Perovskite Solar Cells. Small 2023, 19 (3). https://doi.org/10.1002/smll.202205088.
33. 40. Su, P.; Bai, L.; Bi, H.; Liu, B.; Chen, S.; Lee, D.; Yang, H.; Chen, C.; Zang, Z.; Chen, J. Interfacial Gradient Energy Band Alignment Modulation via Ion Exchange Reaction toward Efficient and Stable Methylammonium-Free Dion-Jacobson Quasi-2D Perovskite Solar Cells. Journal of Power Sources 2021, 506, 230213. https://doi.org/10.1016/j.jpowsour.2021.230213.
34. 67. Lv, G.; Li, L.; Lu, D.; Xu, Z.; Dong, Y.; Li, Q.; Chang, Z.; Yin, W.-J.; Liu, Y. Multiple-Noncovalent-Interaction-Stabilized Layered Dion–Jacobson Perovskite for Efficient Solar Cells. Nano Letters 2021, 21 (13), 5788–5797. https://doi.org/10.1021/acs.nanolett.1c01505.
35. 36. Wang, H.; Qin, Z.; Xie, J.; Zhao, S.; Liu, K.; Guo, X.; Li, G.; Lu, X.; Yan, K.; Xu, J. Efficient Slantwise Aligned Dion–Jacobson Phase Perovskite Solar Cells Based on Trans-1,4-Cyclohexanediamine. Small 2020, 16 (42). https://doi.org/10.1002/smll.202003098.
36. 68. Xu, Z.; Lu, D.; Dong, X.; Chen, M.; Fu, Q.; Liu, Y. Highly Efficient and Stable Dion–Jacobson Perovskite Solar Cells Enabled by Extended π-Conjugation of Organic Spacer. Advanced Materials 2021, 33 (51). https://doi.org/10.1002/adma.202105083.

37. 25. Wang, J.; Lin, D.; Chen, Y.; Luo, S.; Ke, L.; Ren, X.; Cui, S.; Zhang, L.; Li, Z.; Meng, K.; Lin, Y.; Ding, L.; Yuan, Y. Suppressing the Excessive Solvated Phase for Dion–Jacobson Perovskites with Improved Crystallinity and Vertical Orientation. Solar RRL 2020, 4 (11). https://doi.org/10.1002/solr.202000371.
38. 38. Li, Y.; Milić, J. V.; Ummadisingu, A.; Seo, J.-Y.; Im, J.-H.; Kim, H.-S.; Liu, Y.; Dar, M. I.; Zakeeruddin, S. M.; Wang, P.; Hagfeldt, A.; Grätzel, M. Bifunctional Organic Spacers for Formamidinium-Based Hybrid Dion–Jacobson Two-Dimensional Perovskite Solar Cells. Nano Letters 2019, 19 (1), 150–157. https://doi.org/10.1021/acs.nanolett.8b03552.
39. 39. Cohen, B.-E.; Li, Y.; Meng, Q.; Etgar, L. Dion–Jacobson Two-Dimensional Perovskite Solar Cells Based on Benzene Dimethanammonium Cation. Nano Letters 2019, 19 (4), 2588–2597. https://doi.org/10.1021/acs.nanolett.9b00387.
40. 69. Chen, Z.; Liu, M.; Li, Z.; Shi, T.; Yang, Y.; Yip, H.-L.; Cao, Y. Stable Sn/Pb-Based Perovskite Solar Cells with a Coherent 2D/3D Interface. iScience 2018, 9, 337–346. https://doi.org/10.1016/j.isci.2018.11.003.
